# Animal Models of Spinal Cord Injury

**DOI:** 10.3390/biomedicines13061427

**Published:** 2025-06-10

**Authors:** Vladislav E. Sobolev, Yuriy I. Sysoev, Tatiana V. Vyunova, Pavel E. Musienko

**Affiliations:** 1 Sechenov Institute of Evolutionary Physiology and Biochemistry of the Russian Academy of Sciences, Thorez 44, 194223 Saint Petersburg, Russia; vesob@mail.ru; 2 Laboratory of Motor and Visceral Functions Neuromodulation, Pavlov Institute of Physiology of the Russian Academy of Sciences, 199034 Saint Petersburg, Russia; susoyev92@mail.ru; 3 Life Improvement by Future Technologies Center “LIFT”, 121205 Moscow, Russia; p2@list.ru; 4 National Research Centre «Kurchatov Institute», Kurchatov Sq., 2, 123182 Moscow, Russia; 5 Department of Neurobiology, Sirius University of Science and Technology, 1 Olympic Ave., Sirius, 353340 Sochi, Russia; 6 Institute of Translational Biomedicine, Saint Petersburg State University, 7-9 Universitetskaya Emb., 199034 Saint Petersburg, Russia

**Keywords:** spinal cord injury, animal models, contusion, compression, transection, mice, rats, fish, lampreys, sheep, dogs, cats, pigs, monkeys

## Abstract

Spinal cord injury (SCI) is one of the most frequent causes of disability, accompanied by motor and postural impairments, as well as autonomic and behavioural disorders. Since the beginning of the last century, researchers have been developing and refining experimental models of SCI to study pathogenesis and find therapies. Since the beginning of the 20th century, quite a wide range of methods have been developed for contusion and compression injury, complete and partial transection of the spinal cord, and many others. The choice of model subject in such studies was not limited to mammals, but also included amphibians, lampreys, and even fish. Many functional tests have been proposed to assess functional recovery after injury in laboratory animals, ranging from simple rating scales to locomotion kinematics or recording of spinal neuronal activity. This review describes existing models of SCI in most animal species used in neurobiology. Their key characteristics are discussed, which determine the choice of model and model animals depending on the experimental tasks. Each experimental model of SCI has its own advantages and disadvantages determined by species-specific features of spinal cord anatomy and physiology, the speed of recovery from injury, and the ratio of the necrosis zone to the penumbra. The applicability and availability of the proposed methods for assessing the speed and completeness of recovery is also an important factor.

## 1. Introduction

The spinal cord is a complexly organised structural and functional system that serves as a conduit of information between the brain and the periphery. In humans, the spinal cord includes 31 segments, including 8 cervical, 12 thoracic, 5 lumbar, 5 sacral, and 1 coccygeal. In addition to the C1 segment, which has no sensory nerve root, each segment has a pair of dorsal sensory and ventral motor roots that connect to form a mixed spinal nerve. Spinal cord injuries (SCI) are complex medical conditions resulting from damage to the spinal cord. This damage may be caused by a variety of factors, including trauma from motor vehicle accidents and falls, man-made disasters, and criminal incidents, as well as non-traumatic causes such as malignant tumours and degenerative diseases. Statistics from the World Health Organization indicate that over 15 million individuals are living with a spinal cord injury worldwide as of 2023 [1]. Epidemiological observations indicate that the most prevalent form of SCI in humans is blunt trauma. A published report for the year 2022 indicates that there were 3817 cases of head and spinal cord injuries in the United States. As reported by the National SCI Statistical Center, the number of individuals living with SCI in the United States is estimated to be between 305,000 and 388,000, with approximately 18,000 new cases reported annually [2]. Spinal cord injuries have the potential to result in significant morbidity and permanent disability. The financial burden associated with human spinal cord injury (SCI) extends beyond the initial surgical and therapeutic procedures to encompass the costs of subsequent rehabilitation and ongoing care. The financial burden associated with a spinal cord injury (SCI) is significant and persists throughout the individual’s lifetime. The initial hospitalisation and subsequent rehabilitation, along with modifications to the home and vehicle, and the ongoing costs for durable medical equipment, medications, supplies, and personal care, contribute to a substantial financial obligation. In 2003, the mean cost of hospitalisation and rehabilitation for a patient with a spinal cord injury was USD 282,245 [3]. Therefore, spinal cord injuries in humans currently represent a significant biomedical and economic public health issue.

Since every spinal cord level has its own afferent, efferent, and somatic innervation, cervical, thoracic, lumbar, sacral, and coccygeal SCI result in a variety of symptoms. For example, cervical SCI tends to be the most debilitating because the injury can potentially affect the entire body. Complete injury results in quadriplegia, which describes paralysis in both the upper and lower limbs. Thoracic SCI primarily affects sensation in the trunk and abdomen, as well as the muscles of the trunk and chest. As a result, individuals may experience postural and respiratory difficulties. Thoracic SCI may also affect the innervation of important organs, including the lungs, heart, liver, and upper intestinal tract. Lumbar SCI only affects the lower body, so individuals usually have unaffected motor function and sensation in their hands, arms, and trunk. Because individuals with lumbar SCI experience weakness or paralysis in their legs, they may struggle with walking and balance. Also, since bowel and bladder functions are innervated by the bottommost segments of the sacral spinal cord, individuals with nearly any level of SCI are likely to experience bowel and bladder problems. At the very end of the spinal cord is a single coccygeal nerve. This nerve innervates the skin around the tailbone; therefore, pain, discomfort, or complete loss of sensation in the tailbone area are the main hallmarks of this level of SCI. However, because this nerve makes up the lowest level of the spinal cord, individuals should have no motor disturbances and normal sensation throughout most of their bodies.

In order to understand the pathophysiology of spinal cord injuries and to enable adequate evaluation of potential treatments, the need to develop and improve experimental animal models of spinal cord injury arose as early as the early twentieth century. Animal models of spinal cord injury currently continue to be an informative experimental tool for developing new therapies, assessing regeneration and locomotion. The purpose of this proposed review is to provide an overview of current animal experimental models of spinal cord injury. In historical retrospect, animal models have included a large number of different animal species [4] and the use of a wide range of injury models, from partial or complete transection of the spinal cord to contusions of varying severity and compression squeezing [5,6,7,8,9]. It is noteworthy that the rat and mouse are the most prevalent animal species employed in these models, largely due to their cost-effectiveness and accessibility, as well as their translational potential [10]. It is important to note that different injury models contribute to different issues; therefore, each has its own advantages and disadvantages [11].

The proposed review begins with a general description of the main methodological approaches of experimental spinal cord injury. This review considers all animal species currently used in SCI modelling, as well as the anatomical and functional features of their spinal cords. In the context of the diverse range of animal species, this review examines the various methodologies employed to assess functional recovery from spinal cord injury, encompassing behavioural and functional tests, kinematics, and neurophysiology. Finally, the criteria for the objective choice of animal species and spinal cord injury method in accordance with the investigator’s objectives are discussed, as well as future prospects for the use of animal models of spinal cord injury.

## 2. A Brief History of SCI Methods: Basic Approaches to Developing Animal Models

### 2.1. Spinal Cord Contusion

**Figure 1:**
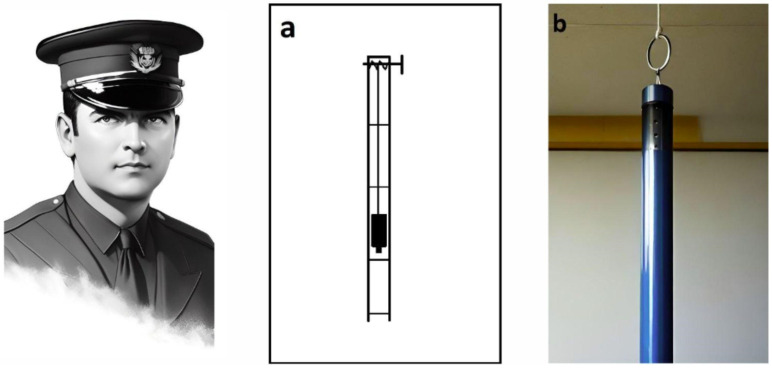
Alfred Reginald Allen (1876–1918) and a schematic of a device (**a**) designed for dosed spinal cord injury in dogs. (**b**). A dream.ai neural network stylised image of Dr Allen’s device based on his textual description. The original drawing is from a 1911 article [13].

One of the first researchers who developed an experimental method of spinal cord injury in animals was Dr. Alfred Reginald Allen (1876–1918) (Figure 1). In 1908, he published a monograph entitled *Spinal Cord Injuries*. Researchers at the time considered it a significant contribution to the understanding of the sequence of pathological events following injury [12]. Dr. Allen’s major scientific achievement was the study of the effects of spinal cord injury, which he began in 1908 and continued until 1914 [13,14].

To simulate mechanical trauma to the spinal cord, Allen developed the ‘Instrument for Obtaining Measured Effects on the Spinal Cord’. The simple load-dropping method used in it is still a widely used model of spinal cord contusion injury [15]. In this case, a load is dropped from a known height through a ventilated guide tube (Figure 1a,b) and strikes a light pressure foot resting on the surface of the dura mater. Upon impact, some of the kinetic energy of the dropped weight is transferred through the pressure foot, causing compression of the spinal cord [15].

Here is how Allen himself describes the use of this device: ‘In my work I have used dogs weighing from 7.5 to 18 kg. The laminectomy was performed in the lower third of the thoracic region. I found that a 30 g weight could be dropped on the spinal cord from a height of not more than 11.5 cm, with complete certainty that the animal would not recover’ [13].

The SCI contusion model developed by Dr. Allen has generated a significant amount of experimental data and modifications of the device continue to be used in the 21st century [15,16,17,18]. If the mass of the load is standardised at 20 g and the impact load is dependent on the height of the fall, there is a pronounced correlation between the severity of the impact and the histological and functional characteristics of the injury sustained [14,15,19,20]. Nevertheless, it was not until another 25 years had elapsed that Dr. Allen’s model began to gain traction in the field of experimental neuroscience. In 1936, Japanese researchers employed it in their investigations into spinal cord injury [21].

**Figure 2:**
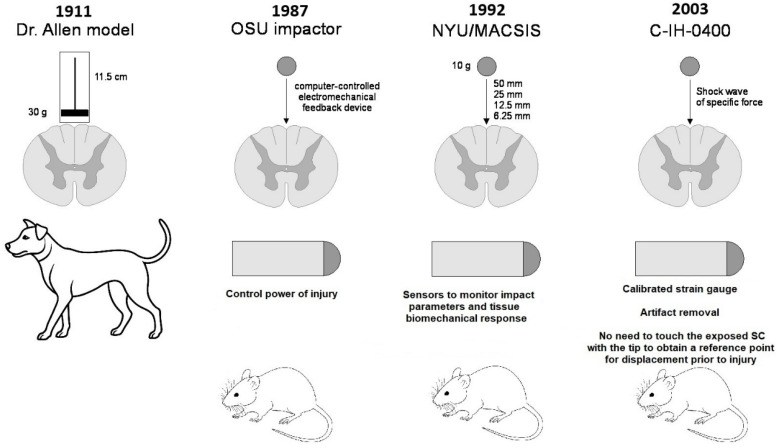
Evolution of SCI contusion animal models.

**Figure 3:**
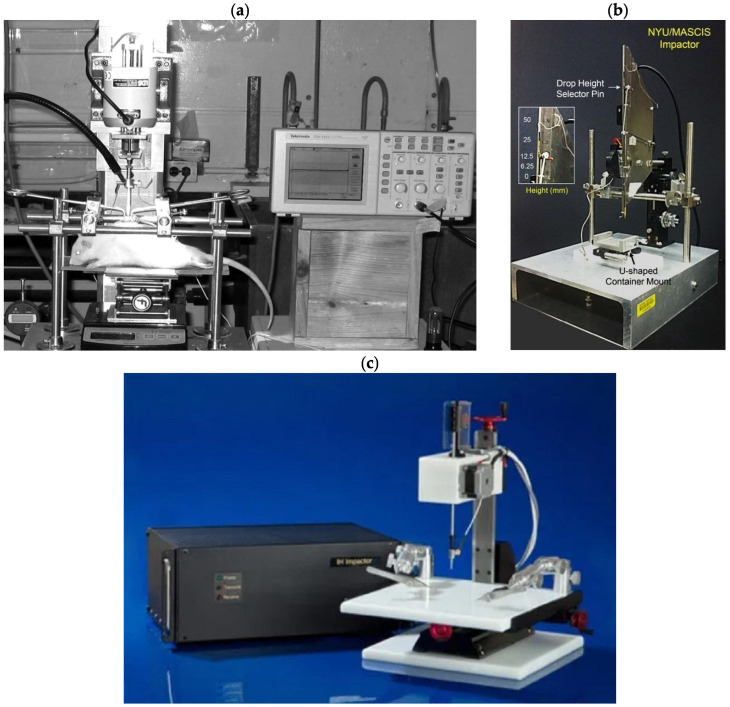
Three types of impactors are (**a**) OSU impactor [27]; (**b**) NYU/MACSIS impactor [28]; and (**c**) IH-0400 impactor (https://psiimpactors.com/product/ih400/) accessed on 15 May 2024. Use permitted under CC BY-NC 4.0.

From 1911 to the present, devices for spinal cord contusion injury in animals have evolved from simple mechanical to modern electronic devices. The latest ones allow control and standardisation of the extent of injury to limit variation between animals and to more adequately compare results obtained in different laboratories. The chronology of development and the most common modern impactors are shown in Figure 2 and Figure 3 [17].

In 1987, a computer-controlled electromechanical feedback device, now known as the OSU Impactor, was developed at Ohio State University to simulate concussion and spinal cord injury in rats (Figure 3a). The device was designed to be sensitive to the characteristics of the injured tissue and to allow continuous control of impact force or tissue displacement [22]. Five years later, at New York University Medical Center, Dr. J.A. Gruner developed a device to simulate SCI with an aggravated contusion equipped with sensors to monitor impact parameters and tissue biomechanical response (Figure 3b). This device, first described in 1992, is now known as the NYU Impactor [23]. This impactor was subsequently refined and improved, and under the name NYU-MASCIS, it was used to standardise the degrees of spinal cord concussion injury caused by dropping a 10 g rod from a height of 6.25 (mild), 12.5 (moderate), 25 (severe), or 50 mm (very severe) onto the exposed dorsal surface of the spinal cord [24].

Another concussion model device was the Infinite Horizon (IH) commercial impactor (Precision Systems & Instrumentation, Lexington, KY, USA), developed in the early 2000s and well proven in rat experiments [25]. This device (Figure 3c) creates a reliable contusion injury to an exposed area of the spinal cord by rapidly delivering a shock wave of a specific force. The principle configuration of the device includes a stepper motor that drives a mechanical shock stand with an attached linear force transducer and shock tip. The force transducer uses a calibrated strain gauge to directly quantify the force generated by the stepper motor and strut on the spinal cord. This eliminates motion artefacts because of the animal breathing. Another notable feature of the IH impactor is that it is not necessary to touch the exposed spinal cord with the tip of the impactor to obtain a reference point for displacement prior to injury. The rack is always connected to the stepper motor, which means that the torque of the motor determines the maximum force level. The offset of the device rack is determined by a linear encoder with a resolution step of 3 µm [26].

### 2.2. Spinal Cord Compression

**Figure 4:**
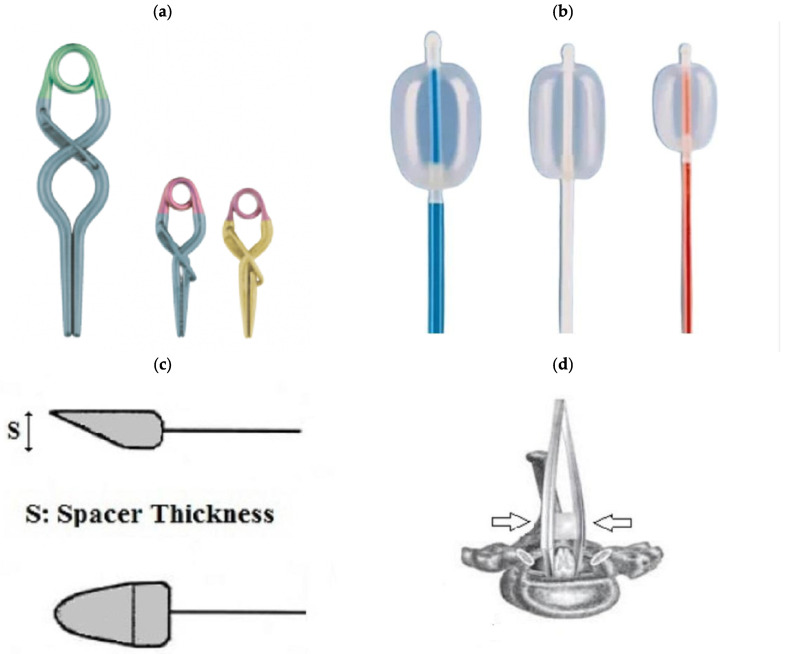
(**a**): Aneurysm clips. (**b**): Fogarty catheter. (**c**): Spacer. (**d**): Calibrated forceps. Arrows indicate the direction of compression. (**c**,**d**) cited by [17]. Use permitted under CC BY-NC 4.0.

Spinal cord compression in animals is used to simulate persistent spinal cord occlusion, which is common in human spinal cord injury. Technically, this variant of spinal cord injury can be modelled using balloons, clips, forceps, screws, or spacers. All of the techniques described below have the same limitation. They all require at least a partial laminectomy to gain access to the spinal cord [6]. Figure 4 shows some device options for modelling compression SCI.

**Figure 5:**
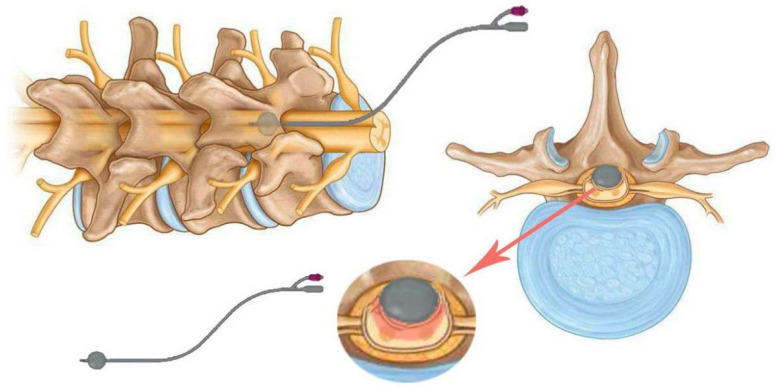
Schematic of the balloon compression model with a 2-French Fogarty catheter and inflatable tip. The catheter is placed under the spine and over the dura mater upstream of the laminectomy and then inflated.

In 1953, a model was created in which the spinal cord of a dog was injured by an inflated balloon inside the spinal canal [29]. The SCI balloon modelling technique requires minimal soft tissue dissection and bone removal. The procedure requires little experimental experience on the part of the operator and can be performed quickly, and the balloon device is easy to handle (Figure 5). In 1957, a method of extradural compression using an inflatable balloon in dogs was proposed [30]. The duration of compression in this method could be precisely monitored and adjusted, but accurate positioning of the balloon was difficult to ensure. In 1973, the Fogarty occlusion catheter was proposed for modelling SCI in cats [31]. Its balloon could be inflated with a known amount of gas, controlling and regulating the duration of spinal cord compression and maintaining its exact location by changing the position of the catheter. Later, in 1975, other researchers used a balloon that could be positioned more precisely. To do this, the spinal cord of dogs was compressed with a cylindrical balloon placed in the T-13 epidural space and a pressure of 160 mmHg was maintained for one hour. This method mimicked spinal cord compression in unrepaired spinal dislocation or fracture-dislocation [32].

**Figure 6:**
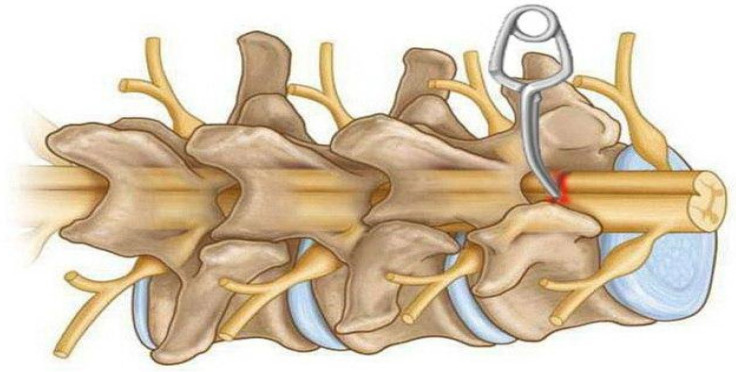
Schematic diagram of a model of spinal cord compression injury using an aneurysmatic clip on the exposed spinal cord after laminectomy. The spinal cord is placed between the two prongs of a special clip, after which the spinal cord is briefly compressed laterally and removed after a short time.

Even before the use of compression clips to model SCI, in 1976, a model of submaximal spinal cord injury using direct compression of the thoracic brain in ferrets was presented [33]. Since then, special calibrated clips have been developed to simulate spinal cord injury due to compression, causing mild, moderate, or severe injury. This method was first proposed in 1978 using a redesigned aneurysm clip to compress the spinal cord of rats with a force of 180 g for different times (Figure 4d). The purpose of this study was to investigate the effect of compression and decompression time on the occurrence of SCI [34]. Subsequently, this method was optimised and used to simulate acute spinal cord compression injury in mice and rats (Figure 6). The procedure most commonly involves thoracic laminectomy followed by the application of clips for a period of 30 s to 1 min to create extradural spinal cord compression [6,35,36].

It is noteworthy that the first comparative study of three methods of experimental modelling of SCI in rats was not conducted until 1983. The methods included the drop weight method, the clip compression method, and the extradural balloon compression method. The findings of the study indicated that both mechanical and vascular factors are involved in the pathogenesis of SCI when clip and balloon compression methods are used [37].

In 1978, a compression model of SCI in rats was developed using clips. In this study, the spinal cord of the animals was compressed for different time intervals with a modified aneurysmal clip with a compression force of 180 g. The results showed a linear relationship between the duration of compression and clinical parameters [34].

In 1991, a model of spinal cord injury in guinea pigs caused by compression to a given thickness was proposed as an alternative to compression or impact with a given force or displacement. The model was technically simple, robust and circumvented some of the biomechanical problems associated with impact technique. It was originally designed to produce moderate injuries, allowing significant recovery of function. A pair of forceps was modified to create an instrument (spacer) for lateral compression of the spinal cord, which is 5 mm long and up to 1.2 mm thick [38]. This model was later standardised for use in rats. To use a spacer, the mean anteroposterior diameter of the spinal canal must first be determined based on the spines of animals of similar weight and age. This makes it possible to determine the spacer size required to obtain an accurate degree of spinal canal narrowing [39].

**Figure 7:**
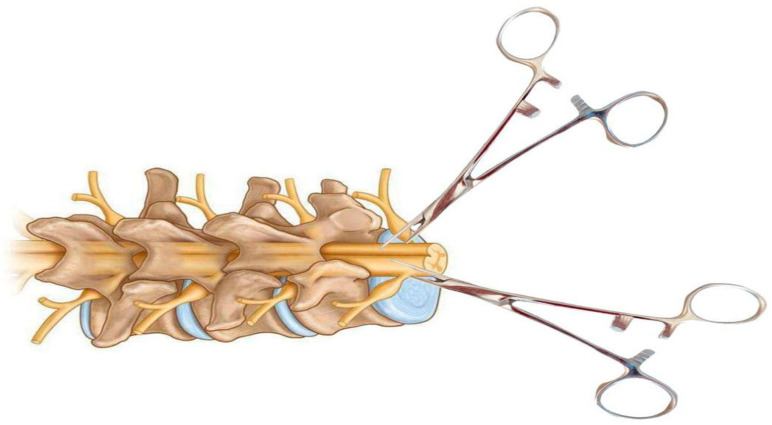
Schematic of a calibrated forceps model for spinal cord compression injury. The forceps are specially modified to include a spacer between the handles for even compression of the spinal cord. This model can be performed in the posterior or lateral plane of the spinal cord after laminectomy.

Another method of spinal cord compression is the use of calibrated forceps and other similar instruments, which have the potential to cause lateral compression injury by exerting pressure on both sides of the spinal cord. This results in the necrosis and displacement of centrally located tissues in the cranial and caudal directions [17]. In 2008, a gradient compression model of SCI was developed in mice using three different forceps with 0.25, 0.4, and 0.55 mm spacers to create lesions of varying severity (Figure 7). Each mouse was subjected to T5–T7 laminectomy, 15 s of spinal cord compression with one of these forceps, behavioural assessment, and post-mortem neuroanatomical analysis [40].

**Figure 8:**
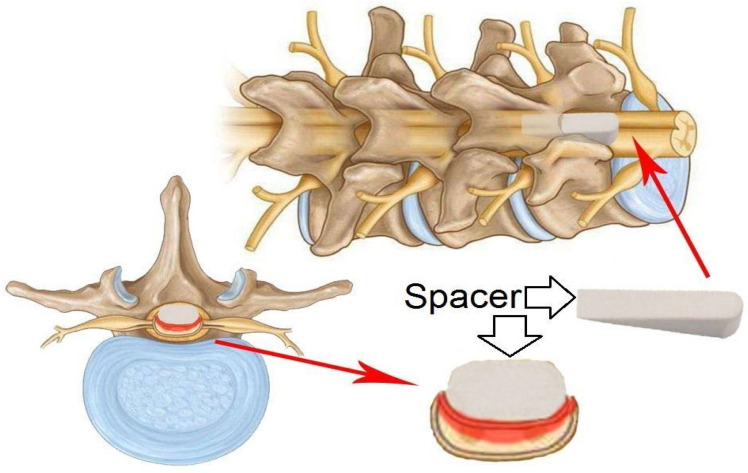
Schematic of a model of a solid wedge-shaped spacer that is inserted between the dorsal vertebral column and the dura mater of the spinal cord after laminectomy. The spacer can be left in place for several days to several weeks.

The objective of solid spacer compression is to simulate compression injuries by inserting a solid, wedge-shaped object into the epidural space (Figure 8).

In this procedure, a laminectomy is performed at the level below that of the desired lesion, after which the spacer is relocated to a position above the dura mater and below the intact lamina [6]. The procedure may result in spinal occlusion at various levels, contingent on the dimensions of the spacer. In a study by Dimar et al., 50% of occlusions were associated with motor deficits. Once inserted, the spacer can remain in the epidural space for an extended period or be surgically removed if necessary [39]. The initial application of this procedure was to test the effects of contusion and compression with a Teflon spacer in a rat model of SCI [39]. This model was subsequently modified to utilise a polymethylmethacrylate or polycarbonate spacer in rats [41,42] and in mice [43].

**Figure 9:**
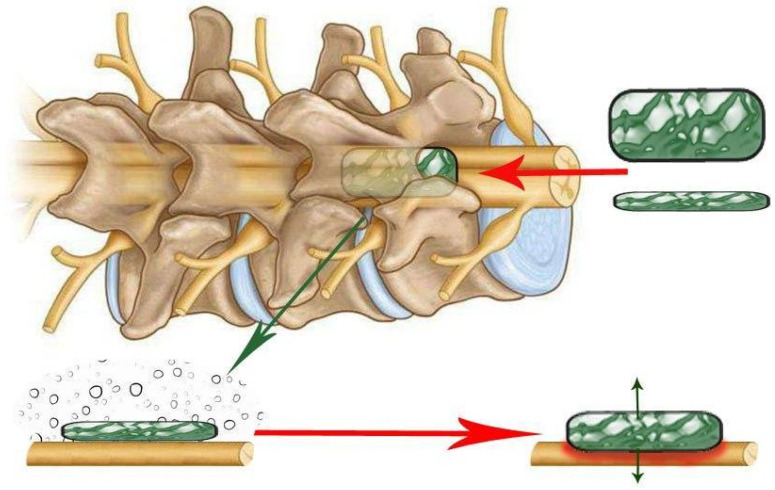
Schematic of an expanding polymer (green rectangle) inserted between the back of the vertebral column and the dura mater of the spinal cord after laminectomy. The polymer fragment expands in place over time, creating a growing spinal cord lesion that slowly compresses the spinal cord.

Another type of SCI is a compressive injury caused by an expanding polymer (Figure 9), as described by Kim et al. In this work, a polymer sheet was used, expanded, and placed between the spinal cord and vertebral column for 25 weeks. This resulted in the creation of a chronic compression injury, which was used to mimic delayed cervical myelopathy. Subsequently, the method has been refined through the utilisation of diverse polymer types and sizes, thereby facilitating the generation of a more precise and reproducible injury [6].

**Figure 10:**
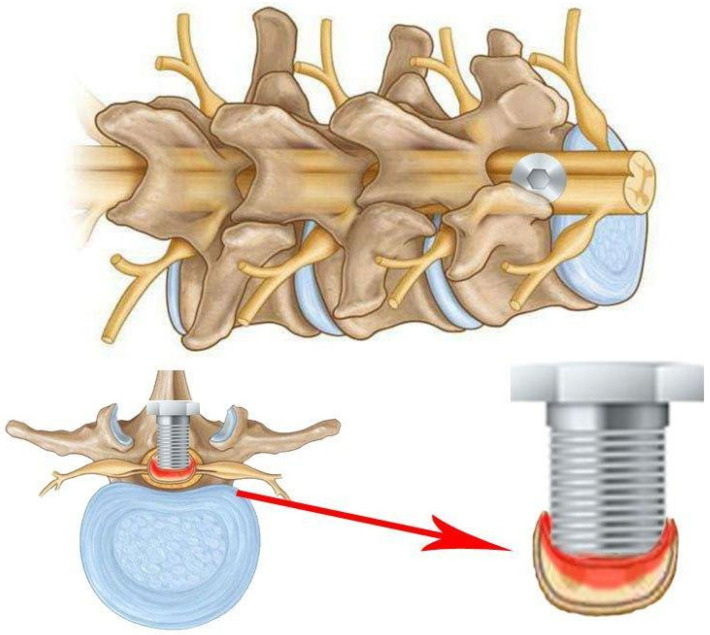
Schematic of the screw method of SCI, in which a screw is passed through the spinal column plate and compresses the spinal cord. The screw can be adjusted gradually over a period of days or weeks.

It is also pertinent to mention compression models of SCI that do not require laminectomy. Of note is the screw compression model (Figure 10), which was first described in 2008 [44]. A small plastic or titanium flat-bottomed screw (0.5 mm pitch, 3 mm diameter, and 9 mm height) is employed for inducing spinal cord injury and is drilled through the lamina in the desired region subsequent to the removal of the spinous process. The screw is then left slightly exposed or sutured until the next procedure. At each interval, the screw is rotated in order to increase compression by 0.1 to 0.5 mm with each rotation. This procedure is typically performed every 7–14 days for a period of 2 months [6,45,46,47,48,49].

This model permits sustained and increasing compression over time, which accurately reflects the morphology of compression entities such as tumours, spondylitis, metastases, and more [45,47]. The procedure’s key benefit is that it does not necessitate the removal of the lamina, thus avoiding any alteration to the hydrodynamics of the spinal cord, which could otherwise impact the outcomes. This is a crucial distinction from other techniques for spinal cord compression, where such alterations may occur [6,44]. Even when the screw is removed, the compression can be reduced by leaving a small portion of the screw to cover the hole in the lamina of the spinal cord [6,44].

**Figure 11:**
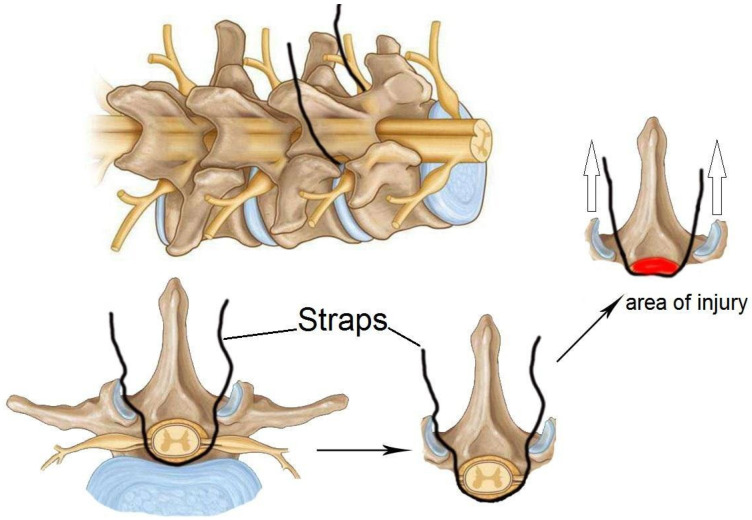
Schematic of a compression injury to the spinal cord using a compression thread or strap. The compression thread or strap passes through the dermal layers and wraps under the spinal cord before exiting the dermis. The threads are then attached to weights that pull the spinal cord dorsally, pressing it against the back of the spinal column. Empty arrows indicate the direction of straps tension.

In 2008, an alternative compression model that does not necessitate laminectomy was proposed: spinal cord strapping [50]. This model results in a minimally invasive spinal cord injury that mimics a spinal cord injury due to increased pressure in the spine (Figure 11).

The model is insufficiently described in the literature, which is likely due to the surgical specificity and complexity of the method. One step of the procedure requires the suture to pass through the dermal layers and between the crevices of the vertebral column. Subsequently, a thread attached to a hooked needle must pass under the spinal cord/dural sheath and exit the dermis without damaging the dura mater and associated blood vessels in order to initiate compression [6,50]. Subsequently, the suture is attached to a pulley system device that has been designed for use in graded trauma, wherein different weights are employed to compress the spinal cord in relation to the posterior spinal column [6,50]. From a surgical perspective, this model is considered minimally invasive and safe when employed in accordance with the relevant guidelines and best practices. The model permits the creation of a gradation of injury that reflects the clinical manifestations of SCI in mild, moderate, and severe cases, while reducing the risk of adverse effects from surgery. However, this model is constrained in its capacity to generate uniform trauma, and its reproducibility remains unvalidated [6,50,51].

### 2.3. Spinal Cord Transection

Clinical cases of complete spinal cord transection in humans are rare, but transection model provide ideal conditions for studying hypotheses about regeneration, degeneration, tissue engineering strategies, and plasticity at the axonal level. The first experiments to assess regeneration in spinal cord transection models were performed in amphibians in the late 19th century. A clear advantage of amphibians over mammals is that adult amphibian neurons have an inherently large growth potential [52]. Axonal regeneration is essential for the recovery of motor function after spinal cord injury. Anatomical and functional regeneration of the spinal cord in amphibians was first described in 1869 in an article in which they observed recovery of motor function in a tailless amphibian larva [53]. The first systematic study of spinal cord regeneration in salamanders at five different stages of development was published in 1955 [54]. In this work, the spinal cord and notochord of two species of salamanders were completely transected and their functional and structural recovery observed at different times ranging from 1 h to 175 days. After simple transection, little cell proliferation and differentiation was observed, suggesting that regeneration consisted mainly of axon regrowth from severed neurons located rostral and caudal to the site of injury [52]. In 1956, researchers observed recovery of function in an axolotl after transection of the spinal cord in the lower trunk [55].

The first experiments to assess functional recovery after spinal cord transection in teleost fish were carried out in the 1920s in the goldfish *Carassius auratus* [56,57]. However, these works were not complemented by significant morphological methods at that time [58]. More detailed observations on the recovery of goldfish [59], guppies (*Lebistes reticulatus*) [60,61], and Japanese rice minnows (*Oryzias latipes*) [62] confirmed that the recovery of swimming behaviour is accompanied by the regeneration of nerve fibres that bridge the gap caused by the spinal cord transection [58,63].

In the 1990s, Danio rerio became another fish species in which spinal cord transection was successfully modelled. Zebrafish (*Danio rerio*) were chosen by researchers as a model to study spinal cord recovery after transection because of their ability to regenerate almost completely. Axonal growth in adult zebrafish reaches functional recovery approximately 4–6 weeks after complete spinal cord transection [64,65,66]. Since 2012, the protocol for spinal cord transection in zebrafish has been standardised and widely used in research [67].

The use of mammals as model animals to assess the effects of complete or partial spinal cord transection also has a long history. As early as 1911–1912, it was shown experimentally that cats exhibited rhythmic alternating contraction of the ankle flexor and extensor muscles following complete transection of the spinal cord and additional dorsal roots [68,69]. In theory, experimental injury by spinal cord transection produces more predictable and standardised clinical and histopathological abnormalities than other methods such as compression or contusion. However, actual experimental results do not always support this conclusion [70]. Depending on the goals of the study, complete or partial spinal cord transection may be performed [7].

Spinal cord transection in laboratory animals is usually performed after laminectomy using thin surgical scissors or a scalpel, which allows targeted destruction of certain conductive pathways, including motor tracts: cortico-spinal tract, rubro-spinal tract or sensory tracts: dorsal columns, or even complete transection of the spinal cord [71]. The model of partial transection (hemisection) of the spinal cord is also popular for studying pathophysiological mechanisms of pain [72]. Since 2015, the rat spinal cord transection model has been standardised and its detailed protocol is published to ensure reproducibility of results [73].

In experiments examining the effects of locomotion, transection is typically conducted at the T8 vertebral level. This lesion preserves the lumbosacral nerves, which control the movement of the legs. Once the transection is completed, the animal is unable to control the bladder. The absence of supraspinal inputs resulting from complete transection provides an advantage for the study of spinal circuits, as it eliminates the potential for interference from other factors [74]. In order to achieve complete motor paralysis without the transection of all spinal cord fibres, it is recommended that a staged hemisection model be employed [75]. In this model, two hemisections are performed at different vertebral levels, such as T7 and T10, on opposite sides of the spinal cord. Reorganisation of circuits in the spared tissue bridge may facilitate repair. Therefore, this well-controlled and reproducible model is an attractive option for studying the mechanisms of recovery after severe injury. The animal care procedures for this model are similar to those described for the complete transection model [74].

In acute SCI models, transection is performed at different brain levels, making it possible to remove the influence of forebrain and higher brainstem centres in order to study the properties and capabilities of the brainstem neural apparatus in the control of posture and locomotion. In particular, transection of the spinal cord (T7–9, T12) allows the study of spinal neural mechanisms proper [9]. Chronic rat models of SCI use complete spinal cord transection at the thoracic level T7–T8 and right lateral hemisection at the cervical level C7, as well as lateral hemisections performed on opposite sides and at different spinal levels (T7 and T1O) [9].

In adult rats, performing a left lateral over-hemisection at the T7 thoracolumbar vertebra and a right lateral hemisection at T10 interrupts all direct supraspinal pathways but leaves a gap of intact tissue. This transection technique results in complete loss of hind limb function, with no evidence of recovery within 2 months of injury. Similarly, individuals with clinically complete SCI often show preservation of connectivity through the lesion. Thus, this experimental lesion replicates key anatomical and functional features of human SCI, while providing a well-controlled environment to study the mechanisms underlying recovery [75].

**Figure 12:**
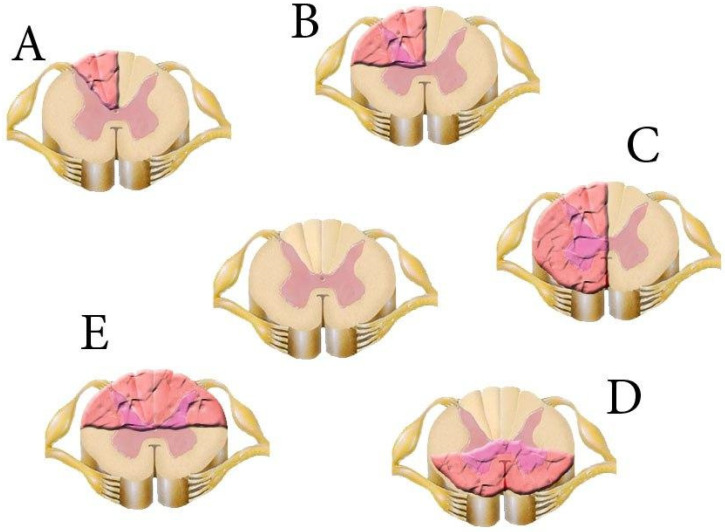
Models of spinal cord transection (pink wavy area). (**A**)—dorsal funiculi transection; (**B**)—dorsolateral funiculus transection; (**C**)—lateral hemisection; (**D**)—ventral column lesion; (**E**)—dorsal column lesion. The intact spinal cord is in the centre.

Partial transections of the spinal cord make it possible to investigate the specific role of descending and ascending projections in locomotor and sensory function, the integration of both systems, and recovery after SCI (Figure 12).

For example, the effects of partial and complete spinal cord transections (Th7–Th8) on EES-evoked locomotor activity in decerebrated cats has been investigated [76]. Transection of the thoracic dorsal half, which contains most of the tracts ascending to the brain stem (afferent fibres of the dorsal columns, tractus spinocerebellaris posterior, the part of tractus spinocerebellaris anterior in the middle of the lateral funiculus, tractus spinocervicalis) [77], did not affect the induction of locomotor rhythmics. This result confirmed that the long spinal–brainstem–spinal loop is not engaged in evoking locomotion by ES. The disruption of dorsal funiculi caused practically no change in the locomotor pattern. Transection of the dorso-lateral funiculus only attenuated the flexor amplitude, most probably because of interruption of the lateral descending motor system (*tractus rubrospinalis, tractus reticulospinalis lateralis*). The obtained data confirmed that the initiation of EES locomotion is caused by direct action on intraspinal systems responsible for locomotor regulation. With intact or partially injured spinal cord, this effect is under the influence of supraspinal motor systems correcting and stabilising the evoked locomotor pattern [76].

The dorsal column lesion of the spinal cord as a model for studying spontaneous recovery has certain advantages, among which, first of all, we can emphasise the possibility to specifically trace the sensory fibres from the sciatic nerve. This makes it possible to perform verifiably complete lesions of the labelled fibres. Although functional deficits from this type of lesion are mild, making assessment of experimental treatment-induced functional recovery difficult, Fagoe et al. proposed a battery of tests in which sensorimotor disturbances can be detected even in the sixth–seventh week after the injury [78].

In the study of Brustein E. and Rossignol S., the authors estimated the recovery of treadmill locomotion of adult cats, subjected to chronic ventral and ventrolateral spinal lesions at low thoracic levels (T11 or T13), preserving at least one dorsolateral funiculus and the dorsal columns [79]. It was shown that all the cats eventually recovered quadrupedal voluntary locomotion despite extensive damage to reticulospinal and vestibulospinal pathways. Initially, in the early period after the spinal lesion (1–3 days for cats with relatively moderate lesions and >3 weeks for cats with most extensive ones, respectively), all the cats suffered from pronounced locomotor and postural deficits, and they could not support their hindquarters or walk with their hindlimbs. Gradually, during the recovery period, they regained quadrupedal walking, although their locomotion was wobbly and inconsistent, and they suffered from poor lateral stability.

To produce complete motor paralysis without transecting all the fibres in the spinal cord, a model of staggered hemisections is recommended. In this model, two hemisections are performed at different vertebral levels, such as T7 and T10, on opposite sides of the spinal cord. The reorganisation of circuits within the spared tissue bridge can support recovery. This well-controlled and reproducible model is, thus, very attractive for the study of the mechanisms of recovery after severe SCI. Other partial lesions, including lateral, dorsal, or ventral hemisection, interrupt specific neural pathways, but because many alternative routes are spared, they are usually followed by an extensive spontaneous recovery that limits the heuristic value of these models for evaluating long-term recovery. During the first few weeks after injury, however, the clear-cut deficits allow the study of the immediate impact of neurotechnologies to alleviate motor deficits. Due to extensive tissue sparing, autonomic functions are usually not impacted, which reduces animal care requirements and improves the animal’s overall well-being [74].

### 2.4. Spinal Cord Photochemical Damage

The model developed in 1986 by Watson B.D. et al. [80] has proven to be one of the most reliable and reproducible experimental models for grading the severity of SCI [81]. The rat spinal cord is exposed by laminectomy and then exposed to 1.5% rose bengal solution (vertebrae T12-L1). Excess dye is washed out with saline and the spinal cord is exposed to “cold” light for 0, 1, 2.5, 5, and 10 min [82]. The resulting photochemical reaction leads to immediate vascular and haemorrhagic necrosis of the central grey matter. On the other hand, an intravascular photochemical reaction occurs by using a dye that is activated by an argon laser to form single oxygen molecules on the endothelial surface of the spinal cord vessels. This leads to a severe platelet reaction, a subsequent vascular occlusion, and parenchymal infarction. The disadvantage of this model is that it is difficult to control the degree of damage to the spinal cord tissue [17,83].

### 2.5. Spinal Cord Ischemic Injury

Spinal cord ischemic injury (SCII) with development of paralysis is a major cause of morbidity after thoracic aortic surgery. Since the vascular supply of the spinal cord is similar in rats and humans, the rat has become important for studying the mechanisms of injury and developing therapeutic strategies to prevent this complication. Kanellopoulos G.K. et al. developed a rat model of SCII in 1997. In this case, the authors induced occlusion of the descending thoracic aorta using an inflated balloon 2F Fogarty catheter inserted through the femoral or left common carotid artery. The combination of aortic arch occlusion and induced hypovolemia provided a reproducible model of SCII in rats [84]. The first description of an ischaemic model of SCII in the mouse was published in 2000 [85]. This model uses an anterior sternotomy with temporary aortic occlusion created by aneurysm clips placed on the aortic arch and left subclavian artery [86].

### 2.6. Spinal Cord Excitotoxic Injury

In 1993, the anatomical, physiological, and behavioural changes associated with the excitotoxic model of SCI were first described [87,88]. Intraspinal injections of the AMPA metabotropic receptor agonist quisqualic acid (QUIS) were used to mimic the injury-induced increase in excitatory amino acid (EAA) levels, a well-documented neurochemical change following spinal cord injury (SCI) [89]. The results showed that different intraspinal QUIS injection strategies, i.e., volume and depth, can produce a gradient pattern of neuronal loss in specific regions of the spinal cord grey matter. This pattern allows specific areas of tissue damage to be correlated with behavioural changes. However, almost all animals develop varying degrees of hypersensitivity to mechanical and thermal stimuli [83].

## 3. Mammalian and Non-Mammalian Animal Models of Spinal Cord Injury

Table 1 presents the most relevant models of spinal cord injury in various animal species, including mammals and non-mammals.

### 3.1. Amphibia Models of Spinal Cord Injury

Tailed amphibians, including salamanders and newts, have a very efficient regenerative capacity [135]. Some species, such as anuran amphibians like *Xenopus laevis*, are able to regenerate SCs during larval stages, but this ability is lost during metamorphosis [136]. Experimental models used to study the response to SCI in salamanders (axolotls and newts) typically involve tail amputation and spinal cord transection [132]. Spinal cord transection is followed by axonal regeneration, neurogenesis, and recovery of near-normal swimming ability within 2–3 months, which depends on the regeneration of descending neurons [131].

The frog *X. laevis* provides a unique experimental animal model to compare recuperative and regenerative responses in the same species [136,137,138]. The pre-metamorphic stages (stage 48–54 NF) show very efficient spinal cord (SC) regeneration and are considered regenerative (R-stages). During metamorphosis (stage 66), this ability is lost, and after metamorphosis, animals, including frogs, are unable to regenerate SC, and are therefore considered regenerative stages (NR-stages) [138]. In 2017, detailed protocols for maintaining *Xenopus laevis* tadpoles and frogs were published, as well as procedures for studying spinal cord regeneration, including methods for modelling SCI, in vivo imaging for cell analysis, a swimming test to measure functional recovery, and a model for screening novel compounds that promote neural regeneration [13]. *X. laevis* is, thus, a unique model organism for studying spinal cord regeneration by comparing the recuperative and regenerative stages of SCI.

### 3.2. Fish Models of Spinal Cord Injury

**Figure 13:**
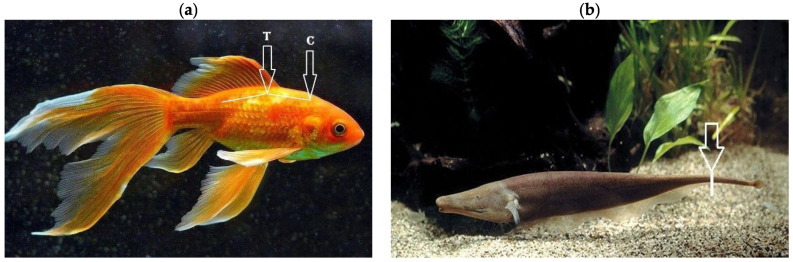
Areas of spinal cord injury modelling in teleost fishes. (**a**) In most fish species, spinal cord transection (line and arrows) is performed at the cervical (C) or thoracic (T) level. (**b**) In *A. leptorhynchus*, caudal spinal cord amputation (line and arrow) can be performed by removing the entire caudal segment of the spinal cord.

Fish as a class of vertebrates have the ability to regenerate the central nervous system after injury in adulthood compared to mammals. In this regard, several fish species have been used as model systems to study spinal cord injury and regeneration [58]. Two main types of spinal cord lesions have been applied to different fish species (Figure 13). The first model involves transection or crushing of the thoracic or cervical spinal cord, resulting mainly in axonal injury. Such injuries require the spinal cord to regrow through a specific gap in the nerve tissue. This model has mainly been used to study axonal regeneration. Studies using spinal cord transection have been carried out in goldfish, zebrafish (*Danio rerio*), minnows *(Phoxinus phoxinus*), guppies, and eels (*Anguilla anguilla*) [58].

The second model involves amputation of the caudal spinal cord, removing the entire section of nerve tissue. Regeneration after such amputations requires complete de novo regrowth and differentiation of different tissue types, including neural tissue. Similar SCI models have only been used in the bony teleost fish Gymnotiformes teleosts, the black ghost fish Apteronotus albifrons, and the brown ghost fish *Apteronotus leptorhynchus* [58,134].

In 2012, a detailed protocol for modelling SCI in zebrafish by complete spinal cord transection was published [67], setting out the necessary pre-experimental parameters of this live model and the tools required. In particular, the authors note that adult Danio rerio should be approximately 6 months old and 2.5–3 cm in length. Health is important for the success of the operation and batches of fish that do not recover well should not be used for further experiments. The number of zebrafish should be calculated before the start of the experiment, taking into account an approximate 70–80% survival rate after surgery [67].

### 3.3. Lamprey Models of Spinal Cord Injury

In 2023, a major review was published, including an analysis of the scientific literature, archival documents and interviews with scientists, detailing the history of lampreys in neurobiology from the 1830s to the present [139]. Lampreys (*Petromyzontidae*) as an animal model of SCI have a number of advantages that make them ideally suited to study the mechanisms that support axonal regeneration and lead to behavioural recovery. The lamprey CNS is favourable for axon growth. Moreover, the lamprey brain contains about 30 large, uniquely identified reticulo-spinal neurons called Müller and Mauthner cells [140]. Following SCI in lampreys, descending brain neurons regenerate their axons and connect to spinal targets caudal to the site of spinal cord injury, resulting in recovery of locomotion and other behavioural functions within weeks [141]. It should be noted that the lamprey CNS shares many features with the nervous systems of higher vertebrates [142,143], while being relatively simple. Modelling SCI in lampreys in this regard allows us to analyse the cellular, synaptic, and integrative properties of the locomotor networks of the brain and spinal cord much more easily than in higher vertebrates.

To investigate the recovery of descending brain and spinal cord projections and locomotor behaviour after SCI, complete spinal cord transections are usually performed at the most rostral levels of the spine. This is necessary to disrupt all downward transmission from the brain’s locomotor command systems to the spinal central pattern generator (JPG) networks. The procedure involves exposing the spinal cord through a dorsal incision and complete transection under microscopic observation at the level of the fifth gill [133]. A comparison of the locomotor behaviour (swimming) and the properties of descending inputs, locomotor networks, and sensory inputs in intact lampreys and in lampreys with complete spinal cord lesions revealed that, in 90% of animals, swimming parameters after lesions recovered to a level similar to that of lesioned animals [14].

### 3.4. Rat Models of Spinal Cord Injury

**Figure 14:**
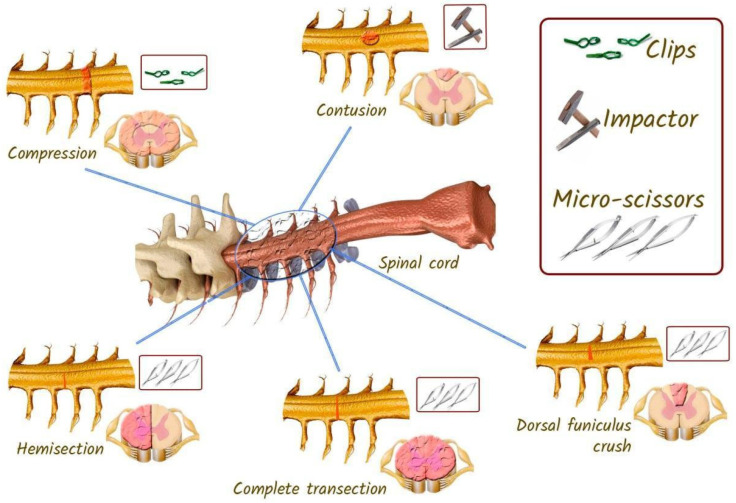
Different models of SCI that can be reproduced in rats. In the compression model, a clamp is used to initiate the injury. In the contusion model of injury, the impactor is dropped from a predetermined distance. In the spinal cord transection model, microsurgical scissors are used.

Laboratory rats are commonly used to assess neuronal recovery after experimental injury. Three different injury models are commonly used in rat experiments with SCI: compression, contusion, and transactional (Figure 14). Among them, contusion and compression are the most common types of injuries encountered in humans [121]. Therefore, to assess neuronal changes and behavioural outcomes, these patterns are very important [18].

The choice of rat line for SCI modelling is an important and relevant factor to consider as it has been shown that morphological, sensory, and motor differences exist between commonly used laboratory rat and mouse lines [144,145,146]. Line choice also influences the development of chronic central pain after spinal cord injury and genuine differences, for example, Sprague-Dawley rats from three different breeders have been found in spontaneous recovery of locomotor functions [147,148].

#### 3.4.1. Rat Spinal Cord Contusion Models

In rats, this thoracic spinal cord injury model is commonly used to assess locomotion and study spinal cord recovery [149]. The injury is usually induced using an impactor [6], which is used to drop a 10 g rod onto the spinal cord [119]. There are currently several types of impactors as described in Section 1. MASCIS uses a computer-controlled 10 g load to induce SCI in rats [23]. After laminectomy, muscles and spinous processes are removed at the desired level of the spine according to the diameter of the impactor. The height of the impactor, its mass and time of fall are preset on the computer before the injury is initiated.

After injury, subdural haemorrhage is usually observed, which can be eliminated by washing with saline solution [119]. Another reliable commercial import is the IH-0400 (Infinite Horizon impactor). It uses system software to apply an impact force to the spinal cord. This injury creates an optimal SCI pattern, reducing variability compared to other existing devices [25].

#### 3.4.2. Rat Spinal Cord Compression Models

This model in rats is best suited for studying therapeutic effects and neuroprotective studies. In addition, this model produces minimal neuronal loss after SCI. The model is also useful for studying secondary damage and cell transplantation therapy [48]. Due to its similarity to traumatic spinal cord injury in humans, the model is also suitable for translational research [121]. In the chronic state, glial scarring forms in the compression injury model, which is very similar to scarring in SCI patients [150].

There are several ways to create a compression model for rats such as calibrated force compression, clip compression, and balloon compression [118]. However, the thoracic level clip compression model correlates much better with functional and histological outcomes [116]. It is an inexpensive technique that uses a specialised clip to compress the spinal cord [117]. Under inhalation anaesthesia, a laminectomy is performed at the desired level of the spine by retracting the muscles and removing the spinous processes and vertebral bodies. A modified aneurysm clamp is then inserted extradurally and held for 60 s, after which the clamp is removed for acute trauma [151]. The muscle and connective tissues are then sutured and the skin is closed. A significant limitation of the compression model is that the resulting damage to neuronal pathways may differ from that intended, which may lead to undesirable results in regeneration studies [121].

#### 3.4.3. Rat Models of Spinal Cord Transection

The models are useful for assessing axonal regeneration and behavioural responses after SCI [152]. The models in question are relatively stable, and the recovery process can be assessed within four weeks of the injury [153]. Two main ligation models are employed in rats: complete and incomplete. In the incomplete transection (hemisection) model, different parts of the spinal cord are excised, including lateral hemisection, dorsal hemisection, or dorsal crush of the funiculus [121]. Complete and incomplete transections are not analogous to clinical SCI in humans and are therefore not considered relevant to human SCI. These models are employed in neuroscience and neuroscience research to investigate neural circuits and pathways [154]. A complete injury necessitates a complete transection of the spinal cord, including the ascending and descending tracts.

To perform a complete transection injury subsequent to laminectomy, microdissection scissors are inserted into the spinal cord with the intention of dissecting it at the desired level and depth. Following the injury, gel foam is injected into the incision site in order to minimise bleeding and confirm the separation of the spinal cord. It is important to note that if a complete transection is not carefully modelled, the ventral portions of the axons may be preserved, resulting in residual motor function of the hind limbs [120]. In the case of an incomplete transection, iridectomy scissors are employed to separate the dorsal and ventral columns of the spinal cord from the lateral to midline, with the tip of the scissors serving as the point of closure [122]. The incisions in the muscle and skin are then sutured, after which the rat is subjected to behavioural tests to assess the efficacy of the therapeutic approaches under study [121].

In order to achieve complete motor paralysis without the transection of all spinal cord fibres, a staged hemisection model has been developed. In this model, two hemisections are performed at distinct vertebral levels, such as T7 and T10, on opposing sides of the spinal cord. The reorganisation of circuits in the spared tissue bridge may facilitate repair [75]. This well-controlled and reproducible model is an attractive option for investigating the mechanisms underlying recovery from severe spinal cord injury [9,74].

### 3.5. Mice Models of Spinal Cord Injury

In mice, SCI models analogous to the rat models described above have been developed and employed, with due consideration given to the mass and anatomical characteristics of this animal species. In general, spinal cord injury is induced in the C- and T-segments of the spinal cord.

In 1998, Kuhn et al. developed a graded contusion model of SCI for mice. The authors employed a falling weight for SC injury, with the experimental groups differing in weight and height. The weight was 2.5 cm, 2 g × 2.5 cm, 3 g × 2.5 cm, and 3 g × 5.0 cm, and the height from which it was dropped onto an impactor resting on the dura mater was 2.5 cm, 5.0 cm, and 7.5 cm. All groups demonstrated significant functional deficits following injury, which were subsequently followed by a gradual recovery. The degree of recovery was found to correlate with the weight lost and the percentage of preserved white matter. The mean percentage of preserved white matter was 41.3 ± 6.0% in the 2 g × 2.5 cm group and 24.3 ± 5.0% in the 3 g × 2.5 cm group [129].

In 2009, Marques et al. developed a simple, reliable, and inexpensive model of clip-assisted spinal cord (SC) compression injury in mice [128]. The model exhibits functional and morphological reproducibility, as well as good validity. C57BL/6 mice were subjected to laminectomy in the T9 region and compression with a vascular clip, exerting a force of 30 g for a period of one minute. Twenty-four hours after the injury, flaccid paralysis was observed in all animals with spinal cord injury (SCI), with subsequent improvement. Morphological analysis of the SCI group in the acute phase revealed the presence of edema, haemorrhage, multiple cavities, fibre degeneration, cell death, and demyelination. In the chronic phase, neuronal death, remyelination of preserved axons, and glial scarring were observed [128].

In 2022, Li et al. [155] described a further simple model of compression FCM in mice using forceps. For this purpose, spinal cord compression was performed in C57BL/6 mice for 3 s, with subsequent observation of regeneration processes for 42 days.

A model of percutaneous compression injury in the cervical spine in mice was developed in 2017 [156]. The authors employed a modified aneurysm clamp to model a bilateral, incomplete injury that closely resembles the FCMs most commonly observed in humans. To achieve this, the spinal cord at the C6 level was compressed for 40 s with a 5.25 g clip. The authors monitored the recovery of injured and falsely operated animals for a period of eight weeks following surgery. Behavioural tests, including the Basso Mouse Scale (BMS), wire suspension, grip strength and automated CatWalk gait analysis, demonstrated that although natural recovery is limited, it occurs to a clinically significant extent during the subacute phase of injury, within 7–14 days after SCI. This study demonstrated that it is feasible to effectively model bilateral cervical spine injury in mice [156].

A model of incomplete spinal cord transection in mice was employed in a randomised and blinded controlled experimental study of exercise-induced locomotion recovery [157]. Following a left-sided hemisection in the T10 region, adult male mice were randomly allocated to either a training or a non-training group. The first group commenced treadmill training one week following surgery and continued for a period of three, six, or nine weeks. Quantitative kinematic gait analysis was employed to evaluate the spatial and temporal characteristics of the left hind limb prior to injury and at 1, 4, 7, or 10 weeks post-injury.

Treadmill training increased stride duration but had a limited effect on hind limb movement pattern. Improvements in trained animals were most evident in the hip and knee joints, while motion recovery in the ankle was limited even after 9 weeks of training. Thus, treadmill training results in only modest improvements in hind limb motion recovery after incomplete SCI in mice [157].

### 3.6. Rabbits Models of Spinal Cord Injury

Published studies on modelling SCI in rabbits have employed contusion, compression, or mixed lesion techniques. In a study published in 2020, the compression model involved spinal decompression of the spinal cord at the T10 level using an aneurysm clamp (REBSTOCK, Dürbheim, Tuttlingen, Germany) at an intensity of 90 g and a holding time of one minute [113]. The authors evaluated the efficacy of the SCI model by examining tail sway and the strength of lower limb contractions in the animals.

In 2007, an intriguing variation in compression SCI in rabbits was proposed, induced by epidural insertion of micro-balloons into the uncovered vertebral column [112]. A midline incision was made at the level of L1–L4, and the paravertebral muscles were dissected bilaterally. A microhemilaminotomy was performed on the right L3 plate, situated in close proximity to the midline, using a Midas-Rex micro diamond drill. The ligamentum flavum was then opened and removed with hydroscissors. A micro-balloon was then inserted into the vertebral column between the bone and dura mater, reaching the level of T12. The micro-balloons were inflated using a specialised device that controlled the pressure and volume. The values of the COE were recorded before and after the injury. Subsequently, the micro-balloon was deflated and completely removed from the epidural space after 15 min. In the postoperative period, all rabbits exhibited paraplegia [112].

To model distraction injury in rabbits, a spinal distractor was created to vary the percentage of distraction by varying the motion between bony landmarks of the spine [115]. Rabbits were operated under anaesthesia to expose vertebral segments from T12 to L4. The distractor was placed on vertebral segments T12 and L4, and displacement was performed by rotating the central screw by 0% (control), 10%, 20%, or 30% of the length from vertebral segments L1 to L4. As the percentage of distraction increased, the severity of SCI increased, as evidenced by neurophysiological testing and biochemical and histopathological changes. According to the authors, the proposed model can be effectively used to study the causes and treatment of distraction injury [115].

A precollicular–postmammillary model was developed to study limb postural reflexes after decerebration in rabbits [157]. The results suggest that the basic mechanisms of postural maintenance and body balance during standing in rabbits are also present in decerebrate animals.

### 3.7. Dog Models of Spinal Cord Injury

Compression models of spinal cord injury (SCI) in dogs have been employed to develop therapeutic approaches [109] or to evaluate sequential histopathological changes in the acute and intermediate phases after injury [110]. In the first study, a 3-Fr embolectomy catheter was inserted into the epidural space through the left hemilaminectomy port in the arch region of the L4 vertebra. The balloons were inflated with 50, 100, or 150 μL of contrast agent at the L1 level for 6, 12, or 24 h, respectively, and spinal canal occlusion (SCO) was measured by computed tomography. The extent of spinal cord injury was assessed using the Albee score, and a histopathological examination was performed 1 week after surgery. The authors found that SCO > 50% at 24 h and >75% at 12 h causes paraplegia within a week after spinal cord injury in dogs [109].

In the second study, an epidural balloon catheter was used to compress the spinal cord of dogs under general anaesthesia for 30 min. An 18-G, 89 mm spinal needle (TOP Corporation, Tokyo, Japan) was utilised, which was inserted into the lumbar epidural space through the lumbosacral joint under fluoroscopic guidance. A guide was inserted through the needle, and following the detection of the guide fluoroscopically, the spinal needle was removed. A 7 Fr intraducer and dilator were inserted into the epidural space with a guide wire. Once the dilator and guide wire had been removed, the intraducer was left in place. The 5 Fr balloon catheter was then inserted into the epidural space through the introducer. The balloon catheter was then advanced under fluoroscopic control to a location between the second and third lumbar vertebrae, after which the balloon was inflated to the desired final volume of 1.0 mL by injecting iohexol using an inflation device into the epidural space for 30 min. The balloon catheter was then deflated and removed [110].

The model of partial spinal cord transection in dogs was employed to investigate the processes of axon regeneration and the potential for stimulation of this process by activated autologous macrophages [111]. The vertebrae T13-L1 were exposed through a midline incision over the shaved and prepared thoracolumbar region. After performing a 2-level laminectomy, complete haemostasis was achieved using a high-speed drill, and the operating microscope was brought to the operative field. In all but one dog (which served as a positive control), a left-sided hemisection of the spinal cord was performed using a sharp-bladed scalpel. The cut ends were separated to create a 5 mm gap. A follow-up of the animals after nine months revealed that there was no histomorphological evidence of axonal regeneration in dogs under the influence of activated autologous macrophages [111].

### 3.8. Cat Models of Spinal Cord Injury

In 1986, Dr Allen’s model of concussive SCI was adapted for cats, and the injury was induced using a weight-dropping apparatus. The vertebral body (T9) below the site of impact was stabilised against displacement with special supports under the transverse processes. The effects of two combinations of weight and height were studied: 10 or 13 g dropped at 20 cm on a 5 mm diameter impact area. Animals were maintained for a period of three to five months following the injury, during which the extent of SC damage was assessed [104]. The number of surviving myelinated axons was found to be dependent on both the weight used and the size of the spinal cord. Impact intensity was determined by calculating the impulse of the weight at impact and dividing it by the cross-sectional area of the spinal cord. At impact intensities greater than 0.02 kg-m/cm/cm^2^, virtually no axonal survival was observed in the centre of the lesion. Between 0.08 and 0.2 kg-m/cm/cm^2^, the number of surviving axons ranged from 100,000 to 2000, approximating a negative exponential function (r = −0.88). The number of axons surviving in the outer 100 µm of the spinal cord exhibited a nearly linear variation (r = −0.82) from a value close to normal to a value of <1% of normal over the same range of injury intensity [104].

The effect of partial and complete transection of the spinal cord (Th7–Th8) on locomotor activity induced in decerebrated cats by electrical epidural stimulation (L5 segment, 80–100 μA, 0.5 ms at 5 Hz) was investigated in cat experiments. Dorsal column transection had no significant effect on locomotion. At the same time, disruption of the ventral quadrant of the spinal cord resulted in the deterioration and instability of locomotor rhythm. Damage to the lateral or medial descending motor systems resulted in redistribution of tone in the antagonist muscles [7].

In 2009, we performed a complete spinal cord dissection in cats at the Th8–Th9 level under ether inhalation and intramuscular injection of 0.3–0.5 mL xylosine; novocaine was injected into the surgical site and 10 min later the spinal cord was dissected, removing a 3–5 mm segment [158]. The aim of the study was to investigate step pattern formation in chronically lamed cats during epidural stimulation (ES). Hind limb stepping performance was dependent on ES parameters and afferent input. At suboptimal ES parameters, no stepping was induced, only muscle reflexes followed the rhythm of stimulation. Optimal ES (20–30 Hz, 150–250 μA for spinal cats) induced coordinated stepping movements in a natural rhythm (0.8–1 Hz) accompanied by electromyographic flash activity of the corresponding muscles [158].

The model of spinal cord transection in cats was used to study the effect of perianal electrical stimulation on frequency-dependent inhibitory and excitatory reflex responses of the bladder [105]. For this purpose, after dorsal laminectomy at the level of T9–T10 vertebrae, local anaesthetic 1% lidocaine was applied to the surface of the spinal cord and then injected subdurally into the spinal cord. After the spinal cord was completely cut, and a piece of foam gel was placed between the cut ends (2–3 mm). The muscle and skin were sutured. Experiments to determine the properties of the spinal reflex from the perianal region to the bladder were performed at least 4–5 weeks after spinal cord surgery. The study showed that activation of pudendal afferent fibres by perianal electrical stimulation can induce frequency dependent bladder reflex responses in cats with chronic SCI, which creates a prerequisite for the development of non-invasive treatment based on perianal electrical stimulation to restore urinary retention and urinary function in people with SCI [105].

### 3.9. Pig Models of Spinal Cord Injury

The use of pigs as a model for studying SCI is becoming increasingly popular among researchers. Therapeutic agents that have demonstrated efficacy in rodent models of SCI have not been successfully employed in human trials, likely due in part to significant anatomical and physiological differences between species [100]. Large animal models represent an attractive intermediate model that may be more successful for translating experimental SCI research into the clinic [159]. Nevertheless, the complexity of care and the range of testing parameters represent significant limitations to modelling SCI in this animal species. It is essential that researchers consider the choice of breed or type of pig, the method of injury, postoperative care, rehabilitation, behavioural outcomes, and histological parameters [160].

A systematic review was published in 2022 in which the authors analysed 1335 full-text English-language articles used in the development of the SCI model in pigs and summarised information on interventions that had been tested using this paradigm [160]. The data analysis yielded 63 studies, of which 33 investigated the pathogenesis of SCI and the remaining 30 investigated other aspects of the modelling and interventions. The mean sample size was 15 pigs with an average weight of 26 kg, and the majority of studies employed female pigs with thoracic spinal cord injury. The most common method of modelling SCI was the dropping of a weight, followed by compression. It is noteworthy that there has been a notable increase in the interest of researchers in this animal species for the purpose of SCI modelling since 2006. Currently, up to eight papers are published per year on this topic.

A range of interventions have been trialled in a porcine model of spinal cord injury (SCI), including increased mean arterial pressure (*n* = 7), electrical stimulation (*n* = 6), stem cell therapy (*n* = 5), hypothermia (*n* = 2), biomaterials (*n* = 2), gene therapy (*n* = 2), steroids (*n* = 1), and nanoparticles (*n* = 1). Consequently, the use of pigs as a model for SCI is a valuable tool for preclinical research [160]. The experiments discussed in the review were conducted using a variety of pig breeds, including both miniature and domestic pigs. A further review, published in 2023, provides a concise overview of the available models of SCI in pigs, outlining their respective capabilities, limitations, and applications [161].

Additionally, SCI modelling in pigs has been conducted on juvenile animals. For instance, a clinically relevant animal model of paediatric SCI was developed in 3–5-month-old piglets. This involved the performance of contusion SCI using a controlled cortical impact at the T7 level [162]. A total of 14 piglets were subjected to complete SCI and 8 to incomplete SCI, after which the recovery of sensorimotor functions was observed. The mean volume of necrotic tissue was found to be greater in the complete SCI group than in the incomplete SCI group. It was not observed that the recovery of sensorimotor functions occurred after complete SCI.

The concussive SCI model has also been successfully employed in domestic pigs, a rather large animal model [100]. A 50 g weight was dropped from a height of either 10 cm (*n* = 3) or 20 cm (*n* = 7) onto the exposed dura mater to induce contusion at the T10 level of the thoracic spine, using a specially designed trauma device. The hind limb motor function was evaluated on days 8 and 13 post-SCI using a 10-point scale. The volume and degree of hyperintensity of the injury-related signal on T2-weighted magnetic resonance (MR) images were assessed on days 3, 7, and 14 after injury. Hind limb motor deficits were observed in all animals 14 days after the spinal cord injury. The animals in the 10 cm group demonstrated some ability to step and transfer body weight and scored an average of 2–3 points higher on a 10-point motor function scale on days 8 and 13 after SCI than the animals in the 20 cm group. The histological lesion volume was 20% larger, and 30% less white matter was affected in the 20 cm group than in the 10 cm group. This study demonstrated the feasibility of graded SCI in the domestic pig, with outcome rates comparable to those observed in models of concussive SCI in miniature pigs [100].

### 3.10. Sheep Models of Spinal Cord Injury

The size and basic anatomical features of the spine and spinal cord in sheep are similar to those of humans [163], and the electrophysiology of their central nervous system has been extensively studied for many years [164]. Sheep can be readily trained to perform a range of tasks on a treadmill, including flexion and extension of the cervical vertebrae [165] and other behavioural tasks. This allows the development of experimental protocols that can provide valuable insights into the pathophysiology of SCI and treatments [99].

The pioneering of spinal cord contusion injury in a sheep model occurred in the 1970s [97,98]. In 2017, the impact drop on the exposed spinal cord from heights of 7.5 and 10 cm was controlled by a linear variable differential transformer (LVDT), thereby refining the methodology. The design of the device is described in detail in Wilson S. et al. (2016) [99]. The surgical procedure involved the exposure of the dorsal portion of the vertebral column at the thoracic T8 level, followed by laminectomy. Subsequently, a weight loss tower was attached to the remaining lateral laminae of the vertebra using bone screws [99]. The authors concluded that the contusion model of SCI in sheep can be used as a suitable model for translational research into new SCS therapies aimed at relieving spasticity in SCI patients.

### 3.11. Primate Models of Spinal Cord Injury

The majority of SCI modelling is conducted on non-human primates (NHPs). The principal objective of modelling SCI in these animals is to replicate the human condition in order to facilitate the development of efficacious treatments. A plethora of experimental models on NHPs exist, with some researchers advocating for the rational allocation of resources to models that are most appropriate for human SCI types [70]. The majority of traumatic spinal cord injuries in humans are the result of blunt trauma, such as motor vehicle collisions or falls.

In a study published in 1976, the authors modelled acute compression SCI in rhesus macaques following laminectomy between T-8 and T-11 using an inflatable extradural cuff. A bespoke apparatus enabled the inflation of the cuff to a pressure of 400 mmHg in 1–2 s [90].

A variety of devices can be employed to simulate spinal cord contusion injury in non-human primates (NHPs), including the Louisville Injury System Apparatus-Large (LISA-L) impact device, which was developed for use in rats [166]. An example of the successful adaptation of this impactor for the modelling of SCI in rhesus macaques (*Macaca mulatta*) was published in 2016 [91]. The authors proceeded to perform a T9 laminectomy, exposing the T10-11 spinal cord segments and removing the yellow ligament while maintaining the integrity of the dura mater. A peak plunger velocity of 1.32 ± 0.05 m/s was achieved using compressed air at 30 psi. The duration of contact between the plunger and the spinal cord was set to 0.25 ± 0.05 s. A 3.2 mm diameter piston tip was used to create a contusion SCI at the level of the T9 vertebra [91].

Additionally, models of spinal cord transection in non-human primates have been employed in non-human primates. This variant of SCI modelling is occasionally subject to criticism on the grounds that the data obtained may not be directly applicable to humans. It is evident that the majority of spinal cord injuries in humans do not entail acute penetrating injuries that result in the opening of the dura mater. For example, in 2012, less than 1% of all spinal cord injuries in the United States were caused by incisional spinal cord injuries [70].

The ethical justifications for models of complete spinal cord excision in primates are challenging to establish [74]. In experiments designed to simulate spinal cord transection in non-human primates (NHPs), injuries are created using sharp instruments such as a scalpel blade or a specialised device [92,93,94]. In certain instances, the outcomes observed do not align with the specifications of conventional SCI models. In particular, spinal cord cuts using standardised lesion creation protocols may result in comparable lesion sizes on histological sections [167]. The surface area of the lesion can vary considerably, from 38% to 95% of the cross-sectional area [167]. It has been observed by some authors that performing accurate and reproducible hemisection in NHP is challenging and uncommon [168]. Nevertheless, several papers have been published reporting the results of qualitative SCI models in NHPs.

In order to ascertain the direct effect of spatiotemporal neuromodulation on walking facilitation in monkeys, a model of lateralised SCI was developed [74]. The lesion is created by making an incision on one side of the thoracic spinal cord, which interrupts the dorsolateral column where the cortico-spinal tract runs. Such an injury results in a temporary paralysis of the ipsilateral leg but does not impair autonomic function or postural control. Significant spontaneous recovery was observed in the animals. A gait that is nearly normal is recovered in the animal approximately four to eight weeks after the injury. A complete lateral hemisection results in a slower and incomplete functional recovery, particularly in cases of cervical SCI [169]. Furthermore, reproducible models of hemicontusional SCI have been developed in non-human primates [170].

In 2012, the results of experiments on the lateral hemisection of the spinal cord at the level of C7 in rhesus macaques (*Macaca mulatta*) were published, following which a subsequent study was conducted on behavioural, electrophysiological, and anatomical parameters [94]. The authors identified significant neuroanatomical and functional differences between rodents and primates that may influence the development of candidate therapeutic drugs. It should be noted, however, that C7 hemisection is similar in nature to the Brown–Séquard injury in humans, which accounts for only 3% of all clinical cases of SCI [171]. Consequently, while this model is useful for evaluating therapies aimed at new axon growth (sprouting or regeneration); it is less relevant than rodent models of contusion for evaluating neuroprotective strategies aimed at improving outcomes after SCI. Monkeys are more expensive to purchase, maintain, and train in detail than rodent studies, perhaps by a factor of 5–10. The authors of the study conclude that the potential benefits and limitations of the model need to be balanced, utilising the primate resource to advance SCI research [94]

Experimental and clinical results show that primates demonstrate faster recovery from lateralized spinal cord injuries compared to symmetrical injuries. To model lateralized spinal cord injury, a comparative study of the effects of lateralized C7 hemisection was performed in monkeys and rats [172]. The results of standardised assessments showed that recovery of locomotion and arm function was faster in monkeys and humans than in rats. Recovery was found to correlate with the formation of corticospinal bypass circuits below the site of injury, which were extensive in monkeys and virtually absent in rats [172].

In 2018, the results of a comparative neuroanatomical analysis of the spinal cord of mice, non-human primates (*Microcebus murinus*) and humans were published [173]. The authors developed and characterised a novel model of lateral spinal cord hemisection in *Microcebus murinus*. A detailed longitudinal behavioural observation was conducted in conjunction with in vivo magnetic resonance imaging (MRI) monitoring for a period of three months following surgery. The distribution of lesions and tissue changes were then compared using three methods: in vivo 1H-MRI, ex vivo 1H-MRI, and classical histology. The overall organisation and distribution/morphology of glial cells in the spinal cord of *M. murinus* was found to be highly comparable to that of humans. A close correlation was observed between the 1H-MRI signal and the reactivity and/or associated post-traumatic phenomena of microglia. The authors conclude that spinal cord hemisection in *M. murinus* represents a novel and reliable model of spinal cord injury (SCI) in non-human primates. This model offers a more accessible alternative to the use of large primates [173].

## 4. Anatomical and Functional Features of the Spinal Cord of Different Animal Species

In a review by Filipp et al., statistical information on publications related to SCI modelling in animals of different species from 1946 to 2018 was presented. Of the 2640 animals experimented on during this period, 1855 (70%) were rats and 444 (16%) were mice. The other animal species were used much less frequently. Consequently, 61 experiments were conducted on rabbits, 56 on dogs, 51 on cats, 42 on pigs, 39 on non-human primates, 15 on guinea pigs, and 77 experiments were on other animal species [174]. Consequently, the statistical data indicates that rats and mice are the most commonly used animals for SCI modelling experiments.

Prior to initiating SCI modelling experiments on animal models, it is prudent for researchers to be cognizant of the distinctions in the structure and functional activity of the spinal cord, not only between species, but even within the same animal species.

### 4.1. Rats and Mice

The anatomical structure of the spinal cord of rats and mice has been well documented for a considerable period of time. Excellent reviews and atlases have been published detailing the structural and functional elements of the spinal cord in rats [175] and in mice [176,177,178]. Nevertheless, the extent of functional recovery following SCI may vary between different animal lines within the same species. For instance, in 2001, the recovery of locomotion and the development of mechanical and thermal allodynia in three commonly used rat strains, namely Long–Evans, Wistar, and Sprague–Dawley, were evaluated using two SCI models [147]. Two models were employed: contusion in the T10 region (NYU impaction, height 12.5 mm) and hemisection in the T13 region. Mechanical stimulation (von Frey filaments) revealed significantly lower baseline responses in Long–Evans rats and significantly higher baseline paw withdrawal latencies during thermal stimulation in Wistar rats compared to other strains. Following contusion injury, the highest percentage of Long–Evans rats (73%) developed mechanical allodynia, while the highest percentage of Sprague–Dawley rats (75%) developed this condition following hemisection injury. It is noteworthy that Sprague–Dawley rats exhibited the highest prevalence (87%) of developing thermal allodynia following brain contusion, whereas 100% of Long–Evans and Sprague–Dawley rats developed thermal allodynia in the hemisection model. The recovery of locomotor function following SCI was comparable across the three models, although Long–Evans rats exhibited a more gradual and limited recovery than the other strains. Sprague–Dawley rats demonstrated faster recovery and greater functional recovery in each model [147].

In 2010, a comprehensive comparative analysis of neuropathological differences between Sprague–Dawley rats and C57Bl6 mice following contusion SCI at the T9 level 28 days after injury was conducted [179]. The researchers employed two magnetic resonance imaging (MRI) protocols for imaging purposes, assessed blood–brain barrier permeability (BBB), performed histological and immunohistochemical studies, and so forth. Consequently, 28 days after injury, both rats and mice exhibited comparable alterations in the anatomy of the spinal cord, including inflammation and glial scar formation. A notable discrepancy was observed in the permeability of the haematospinal barrier, with rats exhibiting a higher degree of permeability than mice. The penetration was more diffuse, extending rostrally and caudally beyond the lesion focus [179].

### 4.2. Mammalian Versus Humans

**Figure 15:**
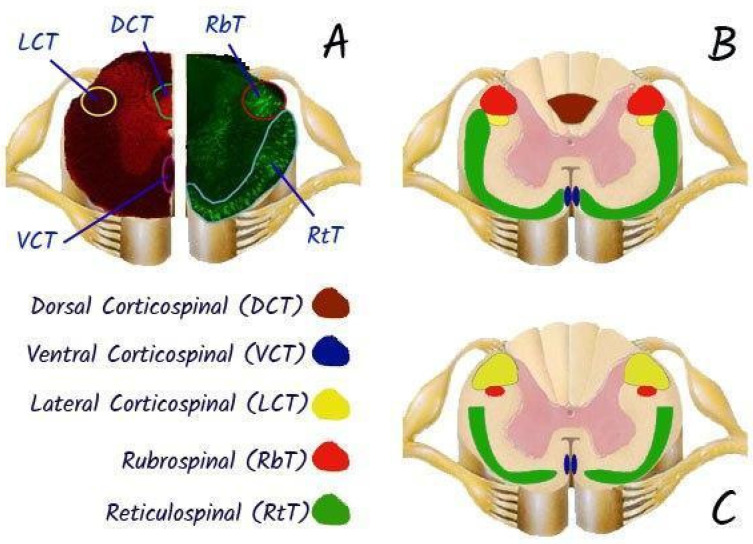
Transverse sections of the rat (**A**,**B**) and human (**C**) demonstrating the approximate locations and sizes of the corticospinal, rubrospinal, and reticulospinal tracts. Rats were injected with an anterograde tracer, biotinylated dextran amine, into the motor cortex (**A**), reticular formation and red nucleus (**B**). Injections performed according to [180]. The corticospinal tract (CST) has larger lateral fibres in humans (yellow), while there is no dorsal CST in humans as compared to rats, who have a large dorsal CST (green). Both rats and humans have a ventral CST (pink). The rubrospinal tract (red) is prominent in rats, but largely reduced in humans, only passing through the upper cervical levels. All species express the reticulospinal tract (blue) prominently, with slight variations in location and size. High-order non-human primates more closely resemble humans; however, tract size and exact location varies between non-human primate species. Adapted from [174]. Use permitted under CC BY-NC 4.0.

The processes of spinal cord reorganisation after SCI have differences between the different animal species used as animal models. In particular, the location, function, and size of the corticospinal tract (CST) play an important role in functional recovery after SCI because it is the major ascending motor pathway from the cerebral cortex to the spinal cord. As shown in Figure 15, the CST has a pronounced dorsal region in rats, while in humans, it has only a lateral and anterior region [174].

The functions of the CST therefore differ between species. In rats, CST axons must synapse on interneurons to relay information to motor neurons because there are no direct connections between the CST and motor neurons [181]. In contrast, in primates, direct cortico-motor neuronal projections eliminate the need for interneuronal connections for forelimb movement [182]. In addition to the development of direct corticospinal connections, an increase in the size and number of corticospinal fibres and excitatory postsynaptic potentials of cortical neurons has been observed in great apes and humans [183]. In great apes, the CST strongly influences the activity of several clusters of motor neurons in the spinal cord, which then innervate the distal muscles of the arms and legs [183]. This is the major control pathway that determines forelimb dexterity and is involved in fine movements of the hands and fingers. In rats, the CST plays a less important role in forelimb movement [184]. In humans, it is also involved in gross and fine motor movements of the hands. Differences in the location and function of the CST in different species are likely to contribute to different degrees of functional recovery [174].

One notable difference between humans and great apes and rats is the presence of the rubrospinal tract. In all these species, the rubrospinal tract is thought to contribute to functional recovery to varying degrees, but in humans, the rubrospinal tract is thought to make a limited contribution to functional recovery. In bipedal species, the rubrospinal tract is reduced, probably due to the increase in CST during the evolutionary transition from quadrupeds to bipeds [185]. Thus, rats, and to some extent non-human primates, show reorganisation of the rubrospinal tract after SCI, whereas humans do not. It is possible that the rubrospinal tract influences functional recovery in humans, particularly after cervical spine trauma, but the extent to which this occurs is unknown [174].

#### 4.2.1. Time of Development of Pathological Changes in the Spinal Cord After Injury

The time course of pathophysiological changes in the spinal cord after SCI varies between species. In humans, the spinal shock phase lasts several weeks or more, whereas in animal models, it lasts only a few hours or days [186]. Given that much of the spontaneous functional recovery is seen within 2–6 months of injury [187], the duration of the spinal shock phase may influence spontaneous functional recovery, but this is usually associated with rehabilitation interventions [174].

A plethora of clinical trials based on successful preclinical studies in SCI models, mainly in rodents, have not led to similarly successful outcomes in humans [188,189,190]. A potentially important but largely neglected factor contributing to these failures is the difference in biological timescales between rodents and humans. Pathological processes, particularly in traumatic spinal cord injury, can change rapidly over time, making it easy to miss the therapeutic window for therapeutic intervention [190]. While some time-scales of biochemical processes, such as enzyme kinetics, may be comparable [191], more complex biological processes, such as metabolic rate, regeneration rate, and lifespan, occur on very different time scales in rodents compared to humans [190]. When it comes to clinically relevant, complex pathologies such as inflammation, “rat/mouse hour” or “rat/mouse day” are not equivalent to “human hour” or “human day”, and vice versa [192].

As the complexity of the biological process increases, so do the differences between the timescales of the two species (Table 2). For example, m/tRNA turnover in rodents (rats) is ~2.5 times faster than in humans, while protein turnover in rodents is ~10 times faster [190,193]. Basal metabolic rate (BMR) is defined as “the minimum rate of energy expenditure per unit time in endothermic animals at rest” [194]. BMR in rats is 8 W/kg compared to 1.25 W/kg in humans; in other words, BMR in rodents is ~6.4 times faster. Heart rate in rats is on average ~4.7 times higher than in humans (260–400 vs. 60–80 beats/min) and the respiratory rate in rats is ~6.3 times higher than in humans (75–115 vs. 12–18/min). Genomic responses in various inflammatory diseases in rodents (mice) are ~30–50 times faster than in humans [192].

Compared to humans, rodents live short and accelerated lives (Table 2). Rats live up to for 3 years (1095 days) [195,196] compared to a human lifespan of ~80 years (29,200 days). Overall, rats live ~27 times faster, meaning that 1 rat day is equivalent to approximately 27 human days, and ~13.5 rat days are equivalent to 1 human year. It is important to note that the time differences between humans and rats depend on the stage of life. For example, gestation in rats is completed within 23 days of conception compared to 280 days in humans, indicating a ~12-fold acceleration of the process. Weaning is completed within 34 days after birth in rats compared to 180 days in humans, a ~5-fold acceleration. Rats live an average of 16 months (486 days) after weaning compared to a human period of 29 years (10,585 days); thus, at this stage of life, one human year is approximately equivalent to 17 rat days [190].

#### 4.2.2. Cellular Responses in the Damaged Spinal Cord Area

The lesion focus of chronic SCI in humans appears to contain fewer reactive astrocytes [197,198] than in rodents [199]. This reduction in astrogliosis may have implications for long-term functional recovery. Although axon sprouting can lead to functional recovery, it can also lead to aberrant connections with potentially deleterious consequences [174]. Pain and autonomic dysreflexia have been associated with increased primary afferent sprouting in animal models [200,201].

Pain, autonomic dysreflexia and spasticity are also common in people with SCI and are likely to be exacerbated in the presence of aberrant connections [202,203,204,205]. In addition, cavity formation [206,207,208] and the slow spread of cell death away from the site of injury [209,210] affect recovery in both humans and animals [174].

#### 4.2.3. Anatomical Features of the Spinal Cord

When designing SCI modelling experiments and selecting the site of injury, it is important to understand the differences in the anatomical structure and size of the spinal cord between the model and the human. For example, in cynomolgus monkeys (*Macaca fascicularis*), the space occupied by cerebrospinal fluid in the cervical vertebrae is statistically significantly smaller than in humans. In humans, the vertebral body, spinal canal and spinal cord are significantly flatter [211]. Knowledge of the morphometric characteristics of the different regions of the spinal cord in animal models is also an important element in experimental design. When planning SCI modelling in an animal model, it is recommended to use spinal anatomical [212], topographical [176], and neurochemical [213] atlases of the spinal cord.

In 2021, the results of a study of the comparative neuroanatomy of the lumbosacral spinal cord of rat, cat, pig, monkey, and human were published [214]. The resulting atlas provides a neuroanatomical reference for the intact lumbosacral spinal cord in these species. The size of the spinal cord segments, the cross-sectional area and the location of the grey and white matter of the spinal cord were quantified and compared between species. The enlargement of the lumbar spinal cord included the spinal cord levels L3-S1 in rats, L4-S1 in cats, L3-S1 in pigs, L2/L3-L7/S1 in monkeys, and T12/L1-S1/S2 in humans. The greatest and most similar increases in size (length and cross-sectional area) are observed in pigs and humans, followed by monkeys and cats, and then rats [214].

Zebrafish share many genetic similarities with humans, but differences in anatomy and physiology may limit the direct applicability of findings. For example, the ability of zebrafish to regenerate whole organs such as the heart [215] is not characteristic of mammals, and this significant difference in regenerative capacity may pose challenges in translating discoveries made in zebrafish into therapeutic regimens for humans [216,217]. The zebrafish spinal cord has a similar structure to that found in all vertebrates. It has dorsal and ventral grey matter regions that correspond to the dorsal and ventral horns in mammals [218]. The spinal cord contains sensory neurons, motor neurons, and a variety of interneurons, including neurons in contact with the central canal, such as the Colmer–Agdur GABAergic cells located in the ependymal layer [219,220]. Interactions between different types of neurons control locomotor behaviour [221]. In the zebrafish embryo, primary motor neurons have a stereotyped position and projection within each segment. They are individually identified by cell body position and axon projection. Secondary neurons, which develop later, are smaller and more numerous [218,222,223].

### 4.3. Differences Between Animal and Human Locomotion

**Figure 16:**
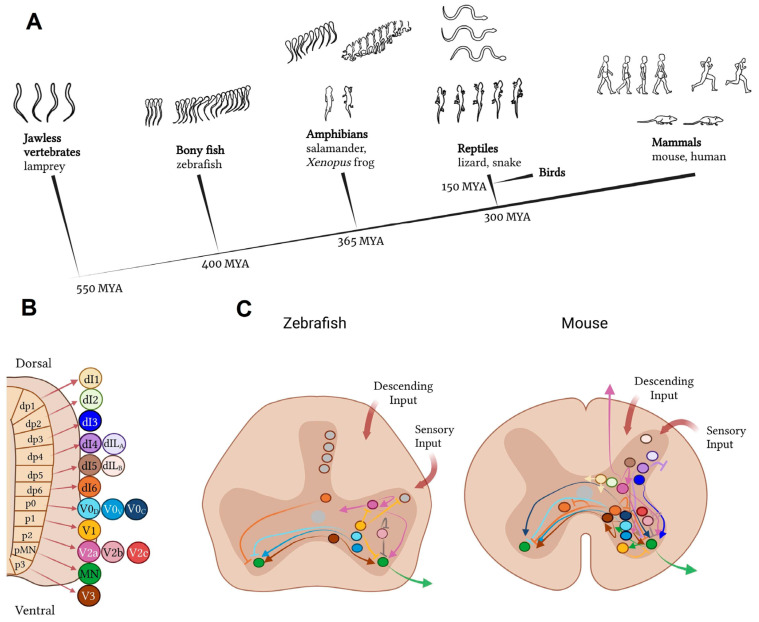
Cross-species comparison of the neural basis of vertebrate movement. (**A**) Cladogram of vertebrate evolution with illustrations of movement patterns for each of the species listed as examples. The lamprey is the most primitive vertebrate and exhibits simple, undulatory swimming; zebrafish display more complex swimming patterns; the frog and salamander use both tail and limbs for movement; reptiles exhibit diagonal limb coordination; and mammals display complex fore−/hindlimb gaits. (**B**) Cardinal neuron classes that make up the spinal cord circuitry are derived from 11 progenitor domains. Some domains give rise to more than one neuron class, e.g., the p2 domain gives rise to the V2a, V2b, and V2c interneurons. (**C**) Comparison of interneuron subtypes and projection patterns in the spinal cord of zebrafish versus mice. Colours represent different neuron classes; grey represents neurons without a clear cardinal class identity [226]. Use permitted under CC BY-NC 4.0.

Vertebrates have a wide range of distinctive locomotor patterns. Over evolutionary time, they have shifted from axial swimming to locomotion using their limbs (Figure 16). Between species, they have uniquely adapted their locomotor repertoires to their environment, physiological needs, and mode of locomotion [224,225,226].

Fish use precise and alternating contraction of segments along the rostrocaudal axis to enable slow, undulating swimming. Mice coordinate limb flexor and extensor muscles to grasp food pellets, run in a wheel, swim, and perform stereotyped repetitive grooming behaviours. Frogs adopt fish-like undulating movements as tadpoles, switch to limb-based locomotion during metamorphosis, and rely preferentially on synchronised limb movements as adults [226,227,228,229,230,231].

This is in contrast to other amphibians, such as salamanders, which maintain both undulating tail movements and limb alternation throughout life. Like salamanders, limbed reptiles and most mammals, including mice and humans, also alternate limb muscles at all speeds as a default behaviour [227,232]. These many differences in basic motor patterns between species should be taken into account when translating data from SCI modelling experiments from animal models to humans [226].

## 5. Methods for Assessing Functional Recovery After Spinal Cord Injury: Behavioural and Functional Tests, Kinematics, Neurophysiology

Table 3 presents the most significant methods for assessing the recovery of motor and postural functions in spinal injury models in various animal species.

In clinical practice, SCI is accompanied by a complex of functional disorders which include motor and sensory disorders, as well as postural instability [285,286,287]. Nearly all currently proposed models of SCI in laboratory animals largely reflect the clinical picture of these disorders and the choice of a particular model organism depends on the experimental objectives and logistical support of the laboratory. Relevant tests have been developed for each group of symptoms, the use of which makes it possible, on the one hand, to study the pathogenesis of primary and secondary lesions in neurotraumas, and, on the other hand, to evaluate the effectiveness of new pharmacological and neurorehabilitation approaches. The key factors that determine the feasibility of particular tests are the size of the animals, their usual habitat (e.g., terrestrial, amphibian, or aquatic), and baseline locomotor activity.

### 5.1. Rat Tests

It is quite expected that the most commonly used species in SCI studies are rodents and, in particular, rats. Their small size and relative ease of maintenance compared to higher mammals or aquatic animals determine their high popularity among researchers. For rats, all sorts of research methods are available, from simple scoring scales [233,234,235,236] to recording of spinal cord neuronal activity. Ahmed et al. [121] reviewed the main behavioural tests to assess motor and sensory function in rats after SCI. The division of tests into motor (skilled forelimb reaching task, IBB, grip strength), locomotor (BBB, open field, KSAT), sensory (Von Frey test, test for hot/cold sensation) and sensorimotor (rung ladder, tapered beam walking) is proposed. In the first case, it is possible to assess the condition of the corticospinal tract, which plays a key role in conscious limb movements. For example, in the skilled forelimb reaching task, the test animal is required to reach pellets lying opposite through a 1–2 cm wide hole. The percentage of “successful” attempts is counted, as well as the time taken [238,239]. In case of SCI at the cervical level, there are pronounced disorders of forelimb function registered within a few weeks after the injury. In this case, the test is proved to be sensitive enough to detect positive therapeutic effects such as epidural electrical stimulation [287]. An alternative to the skilled forelimb reaching task can be the staircase test [288], in which pellets are placed on ladders to the left and right of the tested animal. In general, the ability to assess the “grasping” function of the forelimbs is an important advantage of mice and rats, and this makes them indispensable model subjects when working with SCI at the cervical and upper thoracic levels.

In locomotor tests, the key neuroanatomical substrate is the central pattern generator (CPG), which is located in rats and other mammals primarily in the lumbar region of the spinal cord. The CPG is a neural circuit that, when activated, allows the generation of rhythmic motor patterns such as walking, swimming, or breathing even in the absence of sensory or descending supraspinal innervation [289,290,291]. The most fundamental test for evaluating locomotor function is the Basso, Beattie, and Bresnahan (BBB) scale, which was developed in the mid-1990s and subsequently became the gold standard for quantifying the recovery of rats following a spinal cord injury (SCI) [233]. The scale assesses a range of parameters, including joint mobility, limb plantarflexion, coordination between the fore and hind limbs, paw position during walking, and others. If there are no motor disorders, the rat scores 21 points, with 0 points being awarded if the limb movements are absent. It is worth noting that, despite its universal popularity, the BBB may not be suitable for some models of SCI. Furthermore, a frequent drawback of this scale is the clustering of animals into groups [292]. Another fundamental test for monitoring the recovery of locomotor function is the assessment of distance travelled and average speed in the open field test [243]. However, in the case that the injured animal becomes accustomed to using the forelimbs, misinterpretation of the results is possible. Similarly, such a problem may also be characteristic of swimming tests in which distance swum is assessed. In such cases, potential solutions could be: (1) exclusion of animals that use only their forelimbs from the experimental group; (2) use of scoring scales (BBB, KSAT or LSS); (3) assessment of walking on a track, treadmill or swimming in a pool. The analysis of hind limb kinematics [75] and/or the recording of electromyographic activity of hindlimb muscles [246,247] may also be employed.

In clinical practice, SCI causes not only motor and autonomic disturbances but also, in some cases, neuropathic pain that is poorly controlled by classical analgesics [293]. Similarly, rats with transection or contusion injury of the SC may experience alterations in tactile and temperature sensitivity, manifested as allodynia [294,295]. Chronic pain may be an important factor affecting the rate of spontaneous recovery and the efficacy of therapeutic procedures [296], thus underscoring the importance of controlling sensory function as an aspect of experimental research. It is important to note that no single test can directly measure pain in an animal. Instead, the presence of an unpleasant emotional experience of pain is indirectly indicated by pulling a body part away from the stimulus, decreased ability to move, agitation, frequent grooming of the affected area, or vocalisation during sensory stimulation [297]. The most commonly employed method for assessing sensory function in rats with SCI is the Von Frey test, which is complemented by tests designed to evaluate temperature sensitivity [121]. In the former method, special filaments of varying diameters are employed, with the tips pressed against the sole or the dorsal surface between the first and second metatarsal bones of the hind paws. Paw retraction indicates that the spinal reflex is active; each test is repeated several times at intervals of a few minutes [298]. The hot plate test was proposed over 80 years ago and, in contrast to the tail flick test, is considered to integrate supraspinal pathways. This is because rats with spinal transection do not withdraw their hindlimbs in the hot plate test. Animals are placed on a metal surface that has been heated to an average temperature of 50–55 °C, and they are surrounded by a cylinder. The interval between the placement of the animal on the hot surface and the onset of the behavioural response to nociceptive stimulation (hind paw licking, jumping, hind paw jerking) is recorded [297]. A cold plate test [299] has been proposed as a means of determining sensitivity to cold exposure. Alternatively, the response can be evaluated in vapour cooling tests in which acetone [300] or ethyl chloride [301] is sprayed on the pre-shaved skin of the body region of interest.

Sensorimotor tests are used to assess recovery from SCI and evaluate the state of somatosensory integration, the “heart” of which is the previously mentioned CPG, which provides basic patterns of locomotor rhythm. Its remarkable property is its ability to be reorganised in response to all kinds of sensory signals from supraspinal, spinal, and peripheral structures. In simple terms, the spinal cord is able to adapt the motor pattern in response to feedback from proprioceptors, cutaneous afferents, and sensory organs (vision, hearing, vestibular apparatus) in order to accommodate environmental conditions [302]. Therefore, in tests involving locomotion on a treadmill or free swimming, the “environment” can be considered as “conditionally constant”, and the key system involved is the CPG. In the event of the appearance of obstacles on the “track”, there is a necessity for rapid readjustments that depend on the operation of the sensory systems. As can be concluded from the previous section, SCI is accompanied by pronounced sensory impairments, which determines the relevance of dividing motor tests into locomotor and sensorimotor. Among the latter, the most popular are the Rung Ladder [234,245] and the Tapered Beam Walking [234], which count the number of slips and missteps of the paws during walking to calculate the degree of neurological deficit. The Obstacle Walking Test proposed by Perrot et al. [303] is a promising approach, but to date, there have been no publications using it to assess recovery from SCI.

Given that SCI patients are characterised by postural instability [285,286], an important area of research in animal models is the assessment of static and statokinetic reflexes that regulate body posture. To some extent; information about the state of postural reflexes can be obtained from BBB scale data. For a more detailed assessment, the Postural Instability Test (PIT) can be employed. In this test, rats are held in a position that is nearly vertical and upside down (in a “wheelbarrow”) over a surface that is covered with sandpaper or other rough material, in close proximity to a ruler. The tip of the rat’s nose is aligned with the zero line on the ruler, and the animal is guided forward by gently holding one of the front paws until it takes a step to regain its centre of gravity. The distance between the tip of the nose and the zero line until the rat has taken a step is employed as a measure of quantification [245]. A number of tests have been proposed to assess kinematic and electromyographic responses in response to displacement [304]. However, due to their methodological complexity (e.g., the need to keep rats stationary while pushing on a platform), these tests have not been widely used in SCI studies.

Electrophysiological methods, such as the recording of spontaneous EMG activity during locomotion or swimming, evoked potentials (cortical responses to muscle stimulation, muscle responses to stimulation of the cortex, spinal cord, or individual nerves), and the recording of individual spinal neurons are often employed in conjunction with behavioural tests or independently. EMG recording allows for the verification of the recovery of motor function of the hindlimbs, as well as the assessment of the level of muscle reciprocity and coactivation. It is typically employed in conjunction with kinematic analysis in locomotion and swimming, yielding numerous kinematic and myographic characteristics of locomotion [305,306]. As a consequence of the intercorrelation of the majority of the recorded parameters, it is possible to employ principal component analysis to reduce the dimensionality of the data. Consequently, a number of integrative characteristics are generated, which permit the assessment of the recovery of specific components of locomotion [246].

The analysis of evoked potentials (EP) allows for the assessment of the state of ascending and descending pathways by evaluating the latencies, amplitudes, and shapes of reflex responses in the cerebral cortex during stimulation of muscles, nerves, or skin afferents (somatosensory evoked potentials, SSEPs) or in muscles during stimulation of the cerebral cortex (motor evoked potentials, MEPs) [248,249]. In general, when compared to normal values observed in healthy animals, the amplitudes of SSEP and MEP are decreased, and latencies are increased during the acute period of SCI [250]. The use of chronically implanted myographic and electrocorticographic electrodes allows for the testing of EP in the weeks and months following injury, as this method does not require the regular anaesthesia of the animals. The recovery of motor function observed in behavioural tests correlates with a gradual increase in response amplitudes and a return of latency values to those of healthy animals [250]. Furthermore, the analysis of responses in muscles during epidural stimulation of different parts of the spinal cord also provides information about the functional state of animals after SCI [124].

It has been demonstrated that certain parameters of brain bioelectrical activity in rats may correlate with functional recovery following a SCI. In the study by Pu et al. [251], the sample entropy, trendless fluctuation analysis (DFA), and Kolmogorov complexity were employed. These nonlinear dynamic metrics reflect the complexity of the EEG signal. On the first day following the injury, there was a pronounced decrease in all three parameters. However, during the following days, the values of all three parameters returned to the initial values observed prior to the injury. It is crucial to note that the observed dynamics exhibited a correlation with the BBB scale scores. This allowed the authors to propose that the metrics utilised reflect the processes of neuroplasticity.

### 5.2. Cat Tests

The cat has been a key model subject in the study of brainstem–spinal mechanisms regulating locomotor function [307]. Due to its larger size compared to rodents, it is possible to implant EMG electrodes in a greater number of muscles of the forelimbs and hindlimbs [252,254]. Similarly, the implantation of additional epidural electrodes on the surface of the spinal cord allows for more precise localization than in small animals. In contrast to mice and rats, fewer issues arise in the analysis of kinematics during locomotion due to the mobility of kinematic markers at the hip, knee, shoulder, or elbow joints. Additionally, numerous researchers assess the strength of the limb support response during treadmill walking, which is challenging to accomplish in rodents [254,255]. Consequently, the cat represents a nearly ideal model subject for the assessment of locomotor function during treadmill walking. The disadvantages and limitations are that scoring scales, motor or sensorimotor tests are hardly used with cats with SCI, and swimming in pools is generally unacceptable.

Although some researchers, including Rudolf Magnus, have employed cats in certain experiments to investigate postural tonic reflexes [308], these animals have not become a prevalent subject for experimental postural assessments. Given the current limitations on the use of cats in laboratory research, alternative species such as minipigs offer a viable option. These animals are suitable for most experimental procedures, as evidenced by the availability of similar methods in the literature [102,103,264].

### 5.3. Rabbit Tests

If the cat can be considered as the main model object for studying locomotion on the treadban, the rabbit occupies the same position in the field of studying postural function. The elicitation of postural responses during standing is accomplished through the application of lateral tilts or lateral pushes. In general, the electromyography (EMG) of hindlimb muscles [157,263] and activity of spinal neurons [263,309] are recorded simultaneously with the signals of mechanical sensors during such tests. To assess the recovery of locomotor function in rabbits after SCI, point scales are usually the only one available option [30,258,259,260,261,262].

### 5.4. Lamprey Tests

To assess the degree of recovery of lampreys after SCI, point scales characterising the degree of rehabilitation of locomotor function during free swimming in the pool are employed [265,266,267]. Within weeks, no more than 30–50% of reticulospinal neuron projections are recovered. However, pronounced histomorphological changes remain in the area of injury, including an hourglass-shaped spinal cord, edema in the area of the central canal, and disruption of the cytoarchitectonics of neurons [310]. Concurrently, the swimming pattern of lampreys with transection does not differ from that of healthy animals after 2–3 months [266]. To gain a more comprehensive understanding of their locomotor function, a test was proposed to assess their ability to burrow into sand [266], an innate instinct of many aquatic animals. In contrast to free swimming, burrowing necessitates the movement between two distinct environments (water and sand), each with distinct physical properties. The proposed the scoring system for the burrowing test is as follows: 0—no burrowing occurs, and the body is not covered with sand; 1—the body is covered rostrally up to the seventh gill slit; 2—the body is covered beyond the seventh gill slit, but a large part of the body is still uncovered; 3—most of the body is covered, but the tip of the tail (i.e., the caudal fin) is exposed; 4—full body coverage. In contrast to the free swimming condition, the burying test does not result in a complete recovery. The authors of the technique suggest that this may be due to incomplete recovery of muscle strength or some sensory restructuring. Consequently, this test offers a more comprehensive examination of the recovery of locomotor function in lampreys following complete spinal cord transection. As in mammals, lamprey experiments employ kinematics and EMG analysis during locomotion (free swimming) [265,268].

### 5.5. Danio Rerio Tests

Due to the small size of the zebrafish (*Danio rerio*), it is uncommon to employ any method other than a straightforward calculation of the distance swum in the allotted time [67,274,275,276] or point scales [277,278]. A more comprehensive assessment of locomotor function is possible using the Swim Tunnel system (Loligo Systems), which involves forcing animals to swim against the flow of water [270,271,272,273]. The velocity of the flow can be modified, thereby enabling the evaluation of not only the locomotor aspect of behaviour, but also muscle strength and endurance. Furthermore, an additional oxygen probe and oxygen sensors have been proposed for measuring oxygen consumption during swimming. It is noteworthy that the analysis of swimming kinematics has gained prominence in studies involving zebrafish larvae, as opposed to adult species [282,283]. A startle response test has also been proposed for larvae, in which a swim distance is estimated in the tested individual when the tail tip is touched, vibrated, or fish is flashed with light [281,284]. Vasudevan et al. recorded spinal neuronal activity to confirm that regenerative neurogenesis in the spinal cord of zebrafish produces interneurons capable of integrating into existing locomotor spinal networks [282].

A review of recent publications in the field of SCI research reveals that the choice of animal species has a significant impact on the methodological approach employed during the study. While a variety of behavioural and neurophysiological methods can be employed to assess recovery after injury in small laboratory animals, larger mammals or aquatic species are more suitable for specific research tasks. For instance, it is not possible to assess forelimb grip strength in cats, and it is similarly challenging to study locomotion during swimming in rabbits. It is important to note that the degree of tissue damage in a spinal cord injury (SCI) does not always correspond to a linear impairment of function. This issue was addressed in a review by Fouad et al. [292], and we would like to reiterate this in the present work. For example, even significant damage to the white matter of the spinal cord may not be detected in behavioural tests, while the slightest differences in the preservation of grey matter leads to the separation of experimental animals into subgroups within one group. At the same time, in the case of complete damage of certain tracts, regardless of the mechanisms of the studied treatment, it will no longer be possible to achieve any visible functional recovery. On the other hand, “nonlinearity” may be a consequence of different degrees of adaptive reorganisation of the various remaining pathways, propriospinal connections, and the spinal locomotor generator within the same animal. Such variability arises, for instance, from the different general motor activity of animals and the compensatory motor pattern that a particular animal chooses.

## 6. Criteria for Selecting an Animal Model of Spinal Cord Injury to Assess Motion Recovery, Efficacy of Pharmacotherapy, and Regeneration

When planning studies utilising the spinal cord injury (SCI) as a model for research, it is crucial to have a hypothetical ideal animal model of SCI in mind. The ideal model should meet the following criteria as much as possible [311]:

(1) The model should mimic lesions similar to those observed in clinical settings.

(2) The model should be controllable, reproducible, and stable.

(3) The model should include a simple technique that is easy to learn.

(4) The equipment used to create the model should be simple and allow for quick manipulation.

There are notable distinctions between experimental and clinical SCI. In both cases, the two most common types of injuries are contusion and compression [312]. However, in experimental animals, these injuries most commonly occur dorsally and in the thoracic spine, while in clinical cases, they occur anteriorly and in the cervical spine [311]. In the majority of cases of spinal cord injury (SCI) in humans, the anterior spinal artery, which supplies approximately three-quarters of the spinal cord tissue, is affected, in contrast to the dorsal arteries, which are most commonly affected in experimental SCI [313].

Epidemiological observations indicate that the most prevalent type of SCI in humans is blunt trauma. In a published report for the year 2022, there were 3817 cases of head and spinal cord injuries within the United States. Of these, 92% (3499 cases) involved blunt trauma injuries, while only 5% (205 cases) involved penetrating types of injuries [314]. According to the National SCI Statistical Center, as of 2024, there are currently 305,000 (in the range of 257,000 to 388,000) individuals in the US living with SCI, with 18,000 new injuries reported annually [2]. A statistical analysis of the causes of SCI indicates that 37.5% of cases are vehicle-related, 31.7% are the result of falls, 15.4% are violence-related, 8.0% are sports-related, 3.7% are medical-related, and 3.8% are of another origin. In evaluating the severity of these SCI cases, 47.4% exhibited incomplete tetraplegia, 20.0% exhibited incomplete paraplegia, 12.3% exhibited complete paraplegia, 5% exhibited complete tetraplegia, and normalisation was observed in 19.7% of cases.

Globally, car accidents are the most common cause of SCI, followed by falls [315]. A study conducted in Korea revealed a notable decline in car crash-related SCI cases, which decreased from 65% in 1990–1999 to 41.9% in 2010–2019. Concurrently, there was an increase in fall-related SCI cases, which rose from 24.9% in 1990–1999 to 46.3% in 2010–2019 [316]. A study published in 2023 examined 363 SCI cases in clinic patients. The results indicated that falls were the most common cause (205, 56.5%), followed by transportation (113, 31.1%) [317]. It is postulated that this phenomenon is attributable to the high prevalence of traumatic injury in elderly people, with a significant proportion of cases resulting from slips and falls [318].

Based on the epidemiologic data presented and the causes of SCI in humans, the most significant models in terms of translational relevance are blunt trauma-related SCI models and partially corresponding models of spinal cord contusion and compression injury. A retrospective analysis of the utilisation of animal models of SCI from 1946 to 2018 indicates that the majority of research efforts have been directed towards the development of these specific variants of spinal cord injury. This statistical information, along with the objectives of these studies, is presented in Filipp et al. [174].

**Figure 17:**
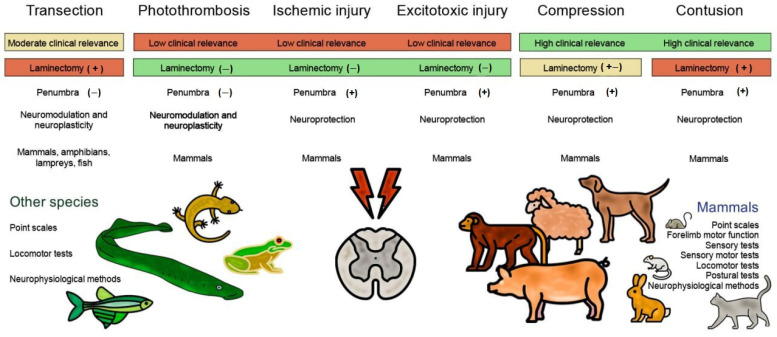
SCI model selection chart. The red color highlights the problematic aspects and methodological difficulties in selecting a specific SCI model. The yellow color highlights the “average balance” between the methodology of SCI models and their clinical significance. Green highlights the more easily implementable SCI models and their correspondence to clinical observations of spinal cord injury in humans. (+) indicates presence in the methodology; (−) indicates absence in the methodology; (+, −) indicates the possibility of both presence and absence in the methodology.

The choice of an animal model of SCI is ultimately determined by several factors, among which we can highlight clinical relevance, the ability to influence the secondary damage cascade, the potential of a neuromodulatory approach, and the speed and completeness of spontaneous recovery. Figure 17 illustrates a simplified algorithm for selecting an animal model of spinal cord injury (SCI) based on the characteristics of each injury type and how closely the model corresponds to human clinical relevance.

When investigating new neuroprotective agents, researchers tend to favour concussion or compression models, while such injuries account for only 40% of cases in clinical practice [319]. Although SCI is caused by dislocation at the cervical level in the majority of patients, it cannot be unequivocally said that any of the proposed animal models is irrelevant. Different models of SCI differ in the severity and temporal dynamics of secondary lesions, which, alone, allows one therapeutic approach to be considered more effective in a particular model than others. In this regard, before starting the study, it is necessary to understand for which patients the developed approach is proposed, and which stages of pathogenesis will be affected.

A cascade of primary and secondary lesions [320] occurs in traumatic SC lesions, resulting in neuronal death and subsequent neurological deficits. The initial signs of primary damage include local haemorrhage, edema, and ischemia, which progress and initiate the secondary phase. This phase is typically distinguished by the presence of acute, subacute, and chronic periods. The acute phase encompasses a number of pathological processes, including spinal shock, vascular dysfunction, ischemic injury, cell membrane disruption, ionic imbalance, and excessive neurotransmitter release. A significant proportion of these events subsequently progress to the subacute phase. The subacute phase is distinguished by the presence of free radicals, lipid peroxidation, and immune-mediated neurotoxicity. Following the conclusion of the initial two phases, the chronic phase is distinguished by the formation of a glial scar that restricts axon sprouting [321]. The application of neuroprotective therapy, which influences the aforementioned processes, has the potential to enhance neuronal survival and reduce neurological deficits [319]. Most studies of neuroprotective agents are conducted in models of SC contusion or compression injury. These models have the advantage of controlled degree of primary injury and significant penumbra volume. The penumbra is the region surrounding the focus of primary injury where a cascade of secondary reactions occurs and is generally believed to have the potential for repair [153]. It is noteworthy that the majority of studies investigating neuroprotective drugs have focused on administration within the first hour following injury. However, in clinical practice, individuals suffering from spinal cord injuries may not be treated until several hours later. Consequently, when working on experimental models, it is crucial to assess and contrast the efficacy of proposed therapies across different time periods [319].

Partial SC transection models are more suitable for investigating axonal regeneration or the formation of new synaptic connections (neuroplasticity) bypassing the site of injury [322,323]. The extent of secondary damage induced by these injury models is more limited, rendering them unsuitable for the assessment of neuroprotective potential. In the mammalian model of complete SC transection, spontaneous locomotion is not restored and can be induced artificially, for example, by epidural electrical stimulation [306]. Spinal locomotor networks are a complex apparatus capable of stepping generation when signals from sensory systems are received. In the absence of descending signals from the brain and brainstem structures in the event of complete damage to the SC, such signals can be transmitted artificially, for example, by mechanical action or electric current [324]. The introduction of drugs that have agonistic or antagonistic effects on receptors of neurotransmitter systems enables the alteration of electromyographic and kinematic characteristics of locomotion, which serves as the foundation for pharmacological neuromodulation therapy [53]. This approach, in conjunction with locomotor training, enables the direction of neuroplasticity processes, which will ultimately enhance the efficacy of neurorehabilitation for individuals with spinal cord injuries [7].

In contrast to humans and other mammals, a number of vertebrates possess the capacity to regenerate the SC following severe injury, including complete rupture. The sprouting of ruptured axons, restoration of neural circuits, and the ability for spontaneous locomotion have been demonstrated in frogs at the tadpole stage, adult salamanders, and to a limited extent in reptiles, lampreys, and bony fish [325]. On the one hand, the use of SCI models in laboratory animals that are incapable of rapid and complete recovery from injury allows the study of new therapeutic approaches in conditions that are close to clinical practice in humans. Conversely, research with fully recuperating species, such as *Danio rerio*, may facilitate the identification of pivotal molecular genetic mechanisms responsible for glial scar formation and the limitation of axon sprouting subsequent to injury. By elucidating these mechanisms, it is anticipated that specific receptor systems may be identified that could be targeted to trigger the process of full regeneration in humans.

## 7. Conclusions and Perspectives

The year 2019 marked the 60th anniversary of the publication of Russell and Birch’s groundbreaking book, *Principles of Humane Experimental Technique*. The 3Rs system they created helped inspire humane progress in scientific practice and is now widely used in experimental biology in in vivo animal models. The 21st century has seen the emergence of promising high-tech non-animal models, such as organs-on-a-chip and computational approaches, which may replace animals as the primary option for biomedical experimentation. The speed of this transition will depend on how quickly these new models are optimised to reflect human biology rather than non-human animals. In this regard, many researchers have called for a three-pronged strategy to (1) develop non-animal methods to replace animal experiments, (2) apply them to biomedical research, and (3) increase their relevance to human biology [326].

Overall, the use of alternative models for research purposes has increased dramatically over the past 25 years, while the use of mammals, with the exception of mice, has decreased or remained stable. From 1990 to 2015, the number of published papers using alternative animals and in silico analyses increased by 909%, compared to 154% for mammals over the same period. One of the most commonly used alternative non-mammalian animal models was the fish Danio rerio. The number of publications using this species increased from a few hundred in 1990 to almost 12,000 in 2011–2015 [327].

The utilisation of guinea pigs and rabbits among mammals has decreased by 68% and 40%, respectively, since 1990, while rats and dogs have been employed on a regular basis for an extended period at a comparable level. Since 2000, there has been a marked upward trend (300.7%) in the number of publications utilising mice as an animal model. Additionally, there has been a notable annual increase in silico modelling, from 7405 studies in 1990 to 88,384 in 2015 [327].

The above statistics illustrate the continued necessity for the utilisation of mammalian animal models, given that current technology is not yet capable of fully replicating the responses of the living system to injury. In this context, it appears that the use of mammalian animal models for SCI modelling is here to stay, although this may change as technology progresses.

The zebrafish, Danio rerio, may be a promising alternative to mammalian animal models for SCI modelling. The use of zebrafish as a model organism offers a distinctive opportunity to investigate the process of neuronal regeneration following a spinal cord injury (SCI). This approach allows for the elucidation of cellular and molecular mechanisms, as well as the identification of potential therapeutic targets [328]. Studies in Danio rerio have already provided valuable information on potential candidate proteins involved in neuroregeneration after SCI, including CTGFa [272], Cav1 [329], ANP32a [330], matrix metalloproteinase-9 [331], and sonic hedgehog [332,333]. In studies of the zebrafish, differentially expressed genes such as the Notch signalling pathway [334,335], the Wnt signalling pathway [336] and the Hippo-Yap/Taz signalling pathway [337] were identified and analysed in detail using bioinformatics. These findings indicate that the molecular mechanisms underlying axon regeneration in adult zebrafish following SCI may be elucidated, and that these candidate genes and pathways may serve as therapeutic targets for the treatment of CNS injury [216,217].

The use of zebrafish as a model for SCI allows for the manipulation of known or unknown genes and proteins, which can then be applied to mammalian SCI models. For example, BDNF is a protein involved in neuronal survival and growth, as well as synaptic plasticity [338,339]. It has been demonstrated to play a role in axon regeneration and functional recovery in zebrafish following SCI [216,217,340]. The overexpression of BDNF in zebrafish has been demonstrated to enhance axonal regeneration and locomotor recovery [341,342]. This evidence suggests that stimulating BDNF expression or signalling may represent a potential therapeutic target for the treatment of SCI in mammals. Similarly, fibroblast growth factors (FGFs) are a family of proteins involved in various cellular processes, including growth, differentiation and tissue repair [343]. In zebrafish, FGF signalling has been demonstrated to play a role in the regenerative response following SCI. The enhancement of FGF signalling has been demonstrated to facilitate axon regeneration, diminish glial scarring, and enhance locomotor recovery in zebrafish models of SCI [278,344]. This finding suggests that modulation of FGF signalling may also be a promising avenue for the treatment of SCI in mammals. The future of SCI research in zebrafish models depends on further intensive study, addressing current challenges and translating the findings [216,217].

In the near future, another promising element in the development and improvement of SCI animal models is likely to be computational modelling of processes using neural networks and artificial intelligence. In this context, computational models have already proven to be a valuable tool for elucidating the function of interneurons in rhythm generation in a range of animal species [226]. They can elucidate experimental observations by testing the minimal requirements for reproducing these observations in silico. Furthermore, computational models can assist in formulating hypotheses regarding the potential alterations in neuronal configuration that may result in disparate outcomes contingent upon developmental stages, external stimuli, or species, and in the event of damage [226].

In conclusion, it is evident that animal models of SCI have proven to be a valuable tool in the understanding of injury mechanisms and regeneration, as well as in the development of experimental treatments. It is clear that animal models will continue to play an important role in SCI-related research, and that their value will only increase in the future.

## Figures and Tables

**Table 1:** Spinal cord injury (SCI) modelling in different animal species.

Class	Order/Species		Type of SCI		Time to Recovery of Spontaneous Locomotion or Swimming	References
Mammals	Primates	Rhesus monkey (Macaca mulatta)	Compression		Several weeks to several months, depending on the severity of the injury. No recovery occurs with complete transection or severe compression/contraction	[90]
			Contusion			[91]
			Complete transection			[92]
			Partial transection	Hemisection		[93,94]
				Dorsal funiculus		[95]
				Lateral funiculus (mainly corticospinal tract)		[96]
	Artiodactyl	Sheep (Ovis aries)	Contusion			[97,98,99]
		Pig (Sus domesticus)	Contusion			[100]
		Minipig	Contusion			[101,102,103]
	Predators	Cat (Felis catus)	Contusion			[104]
			Complete transection			[105,106,107]
			Partial transection	Dorsal funiculus		[76]
				Dorsal funiculus + dorsolateral funiculus		[76]
				ventral column		[79,108]
		Dog (Canis familiaris)	Compression			[109,110]
			Partial transection			[111]
	Lagomorha	Rabbit (Oryctolagus)	Compression			[112,113]
			Contusion			[114]
			Distraction			[115]
	Rodents	Rat (Rattus norvegicus)	Compression			[116,117,118]
			Contusion			[25,119]
			Complete transection			[120,121,122,123]
			Partial transection	Over-hemisection		[123,124]
				Staggered hemisection (two hemisections at different vertebral levels)		[74,75,123,124,125]
				Dorsal column section		[78,126]
				Ventral lesions		[127]
		Mouse (Mus musculus)	Compression			[128]
			Contusion			[129]
			Transection (complete or partial)			[130]
Amphibians	Salamander		Tail amputation or complete transection		2–3 months	[131,132]
	African clawed frog (Xenopus laevis)				20–30 days	
Lampreys	Sea lamprey (*Petromyzon marinus*)		Complete transection at the most rostral levels of the spine		2–3 months	[133]
Fish	Gold fish (*Carassius auratus*) Zebrafish (*Danio rerio*) Eurasian minnow (Phoxinus phoxinus) Guppie (*Poecilia reticulata)* Eel (Anguilla anguilla)		Complete transection		1–2 months	[58,67,134]
	Black ghost knifefish (*Apteronotus albifrons)* Brown ghost knifefish (*A. leptorhynchus)*		Caudal amputation			

**Table 2:** Timelines of basic biological processes in the human and in the rat (*Rattus Norvegicus*) [190].

	Human	Rattus Norvegicus	Times Faster in Rat	One Human Year ≈ Rat Days	One Human Day ≈ Rat Hours	One Human Hour ≈ Rat Minutes
m/tRNA turnover	0.8/day/kg	2/day/kg	2.5			
Protein turnover	1.25/day/kg	12/day/kg	9.6			
Metabolic rate	1.25 W/kg	8 W/kg	6.4			
Heart rate	60–80	260–400	4.7			
Respiratory rate	12–18	75–115	6.3			
Gestation	280 days	21–23 days	12.7	28.7	1.9	4.7
Weaning	180 days	21 days	8.6	42.6	2.8	7
Reaching sexual maturity	4197 days	50 days	84	4.3	0.3	0.8
Reaching adulthood	7300 (20 years)	210 days	35	10.5	0.7	1.7
Reaching reproductive senescence (females)	18,615 days (51 years)	532 days (1.6 years)	35	10.4	0.7	1.7
Post-senescence	10,585 days (29 years)	486 days	22	16.8	1.1	2.7
Life span	29,200 days (80 years)	1095 days (3 years)	26.7	13.7	0.9	2.3

**Table 3:** Methods for assessing locomotor and postural function recovery in SCI models in different animal species.

Animal Species	Method of Analysis	Analysed Parameters	Periods After SCI	Possibility of Multiple Testing	References
Mammals					
Rat/mice	Point scales				
	Basso, Beattie, and Bresnahan Scale (BBB)	Points characterising motor function of fore and hind limbs	Days to weeks after SCI	Yes	[233,234]
	Irvine, Beatties, and Bresnahan (IBB)	Points characterising motor function of forelimbs			[235]
	Karolinska Institutet Swim Assessment Tool (KSAT)	Points characterising motor function in swimming			[236]
	Louisville Swimming Scale (LSS)				[237]
	Tests to assess motor function of the forelimbs				
	Skilled forelimb reaching	Success rate, pellet pulling time	Days to weeks after SCI	Yes	[238,239]
	Grip strength	Grip strength of the forelimbs			[240]
	Sensory tests				
	Von Frey test	Minimum reaction threshold causing paw retraction	Days to weeks after SCI	Yes	[241]
	Assessment of temperature sensitivity (hot/cold plate, tail twitching, etc.).	Threshold temperature eliciting response or pain response score in points			[241]
	Sensorimotor tests				
	Rung ladder	Number of correct paw placements on the bars, number of slips, misses, etc.	Days to weeks after SCI	Yes	[234,242]
	Tapered beam walking	Number of missteps and slips			[234]
	Locomotor tests				
	Open field	Distance travelled, number of freezes, average speed, etc.	Days to weeks after SCI	Yes	[243]
	Analysis of the kinematics of walking on the treadban	Hind limb joint angles, stride length, stride duration, etc.			[75]
	Analysis of swimming kinematics				[244]
	Postural tests				
	Postural instability test (PIT)	Offset required to initiate a step			[245]
	Neurophysiological methods				
	Electromyography during walking/swimming	EMG bursts duration and amplitude, area under the curve, inter-bursts interval, level of reciprocity of muscle pairs, etc.	Days to weeks after SCI	Yes	[246,247]
	Evoked potentials (SSEP, motor cortex and SC stimulation)	Shape of evoked potentials, their latency and amplitude			[248,249,250]
	EEG/ECoG	Sample entropy, detrended fluctuation analysis (DFA), Kolmogorov complexity index			[251]
Cat	Analysis of the kinematics of walking on the treadban	Hind limb joint angles, stride length, duration of swing and stance, etc.	Days to weeks after SCI	Yes	[106,252,253]
	Electromyography in locomotion	Duration and amplitude of EMG bursts, etc.			[252,254]
	Support reaction force	Support reaction force when walking on the treadban			[254,255]
	Evoked potentials (SSEP, motor cortex, and SC stimulation)	Shape of evoked potentials, their latency and amplitude			[256,257]
Rabbit	Point scales for assessing the degree of motor deficits (Zivin et al., Drummond and Moore, Tarlov, Johnson et al.)	Points characterising motor function	Days to weeks after SCI	Yes	[30,258,259,260,261,262]
	Electromyography in postural tests on an inclined platform	Evaluation of the types (correct, incorrect, correct/incorrect, no response) of EMG responses to platform tilt, the ratio of these types, etc.		Yes	[157,263]
	Registration of spinal neuron activity in postural tests on an inclined platform	Average frequency, batch frequency, inter-batch frequency, etc.		No	[263]
Mini-pig	Porcine Thoracic Injury Behavioural Scale	Points characterising motor function	Days to weeks after SCI	Yes	[101,102,103]
	Analysis of the kinematics of walking on the treadban	Hind limb joint angles, stride length, duration of swing and stance phases, etc.			[102,103]
	Electromyography in locomotion	Duration and amplitude of EMG bursts, etc.			[264]
	Evoked potentials (SVEP, motor and during stimulation of the SC or n.sciaticus)	Shape of evoked potentials, their latency and amplitude			[102,103]
Other species					
Lamprey	Free swimming	Points characterising locomotion during swimming	Days to weeks after SCI	Yes	[265,266,267]
	Electromyography	Number of locomotor cycles, intersegmental rostrocaudal phase delays, etc.			[265]
	Evaluation of the ability to burrow into sand	Points characterising the completeness of burial			[266]
	Analysis of swimming kinematics	Maximum amplitude of deviation from the midline, frequency of locomotor movements, etc.			[268]
	Intracellular neuronal registration	Resting potential, action potential amplitudes, firing pattern, etc.		No	[269]
Danio rerio (adult)	Analysis of swimming in the Swim Tunnel (Loligo Systems)	Swimming duration and maximum speed	Days to weeks after SCI	Yes	[270,271,272,273]
	Analysis of free swimming in the aquarium	Distance swum, number and duration of freezes, average speed, etc.		Yes	[67,274,275,276]
	Assessment of locomotor function using point scales	Points characterising locomotion in free swimming		Yes	[277,278]
Danio rerio (larvae)	Assessment of locomotor activity in the Daniovision system (Noldus Information Technology) or ZebraLab Videotrack software (ViewPoint Life Sciences).	Distance swum, number and duration of freezes, average speed, etc.	24–48 h.	Yes	[279,280,281]
	Analysis of swimming kinematics	Maximum angles of inclination between body points, angular velocities, etc.	2–9 days	Yes	[282,283]
	Startle reflex triggered by tail tip touch, vibration, or flash of light	Distance swum	12–72 h.	Yes	[281,284]
	Local fixation of the spinal neuron potential	Evaluation of the correlation of motor activity with neuronal activity	3–9 days	No	[282]

## Data Availability

Not applicable.

## Abbreviations

BBB	Blood–brain barrier
BBB	Basso, Beattie, and Bresnahan scale
BMS	Basso Mouse Scale
CNS	Central nervous system
CPG	Central pattern generator
CST	Corticospinal tract
DCT	Dorsal corticospinal
DFA	Detrended fluctuation analysis
EEG	Electroencephalography
EMG	Electromyography
EP	Evoked potentials
ES	Epidural stimulation
LCT	Lateral corticospinal
LVDT	Linear variable differential transformer
MEPs	Motor evoked potentials
MR	Magnetic resonance
MRI	Magnetic resonance imaging
NHPs	Non-human primates
RbT	Rubrospinal
RtT	Reticulospinal
SC	Spinal cord
SCI	Spinal cord injury
SSEPs	Somatosensory evoked potentials
